# Dynamic rhenium dopant boosts ruthenium oxide for durable oxygen evolution

**DOI:** 10.1038/s41467-023-35913-6

**Published:** 2023-01-21

**Authors:** Huanyu Jin, Xinyan Liu, Pengfei An, Cheng Tang, Huimin Yu, Qinghua Zhang, Hong-Jie Peng, Lin Gu, Yao Zheng, Taeseup Song, Kenneth Davey, Ungyu Paik, Juncai Dong, Shi-Zhang Qiao

**Affiliations:** 1 School of Chemical Engineering and Advanced Materials, The University of Adelaide, Adelaide, SA 5005 Australia; 2 Institute for Sustainability, Energy and Resources, The University of Adelaide, Adelaide, SA 5005 Australia; 3 Institute of Fundamental and Frontier Sciences, University of Electronic Science and Technology of China, Chengdu, 611731 Sichuan China; 4 Beijing Synchrotron Radiation Facility, Institute of High Energy Physics, Chinese Academy of Sciences, Beijing, 100049 China; 5 Future Industries Institute, University of South Australia, Mawson Lakes Campus, Adelaide, SA 5095 Australia; 6 Beijing National Laboratory for Condensed Matter Physics, Institute of Physics, Chinese Academy of Sciences, Beijing, 100190 China; 7 Department of Energy Engineering, Hanyang University, Seoul, 04763 Republic of Korea

**Keywords:** Electrocatalysis, Electrocatalysis, Electrocatalysis, Energy

## Abstract

Heteroatom-doping is a practical means to boost RuO_2_ for acidic oxygen evolution reaction (OER). However, a major drawback is conventional dopants have static electron redistribution. Here, we report that Re dopants in Re_0.06_Ru_0.94_O_2_ undergo a dynamic electron accepting-donating that adaptively boosts activity and stability, which is different from conventional dopants with static dopant electron redistribution. We show Re dopants during OER, (1) accept electrons at the on-site potential to activate Ru site, and (2) donate electrons back at large overpotential and prevent Ru dissolution. We confirm via in situ characterizations and first-principle computation that the dynamic electron-interaction between Re and Ru facilitates the adsorbate evolution mechanism and lowers adsorption energies for oxygen intermediates to boost activity and stability of Re_0.06_Ru_0.94_O_2_. We demonstrate a high mass activity of 500 A g_cata._^−1^ (7811 A g_Re-Ru_^−1^) and a high stability number of S-number = 4.0 × 10^6^ n_oxygen_ n_Ru_^−1^ to outperform most electrocatalysts. We conclude that dynamic dopants can be used to boost activity and stability of active sites and therefore guide the design of adaptive electrocatalysts for clean energy conversions.

## Abstract (web summary)

RuO_2_ is a promising anode catalyst for proton exchange membrane water electrolyzers but suffers from poor catalytic stability. Here the authors present a rhenium-doped RuO_2_ with a unique dynamic electron accepting-donating that adaptively boosts activity and stability in acidic water oxidation.

## Introduction

Proton exchange membrane (PEM) water electrolyzers are practically promising for green hydrogen production because of the high current density, energy efficiency, and safety^1–4^. However, sustainable application is hindered by high price and low reserve of iridium (Ir), the anode material for oxygen evolution reaction (OER)^4–6^. For example, the demand for Ir is nine times greater than current global production for all planned PEM electrolyzers for 2030 in the *Net Zero Emissions Scenario*^1^. Therefore, efficient and Ir-free OER electrocatalysts are important for continuing competitiveness of PEM water electrolyzers^7^.

In 1987 researchers reported that rutile-phase ruthenium oxide (RuO_2_) has significant practical potential to replace IrO_2_ for acidic OER because of excellent activity^8^. However, achieving high activity and stability with this catalyst is practically difficult because of the instability of surface Ru sites in RuO_2_^9–11^. Ru active sites are damaged in two ways, (1) formation of lattice-oxygen vacancies via lattice-oxygen-mediated mechanism (LOM)^12–14^, which leads to instability of the oxygen anion^15^, and (2) over-oxidation of Ru atoms to soluble RuO_4_ species at the high overpotential that leads to demetallation of the active sites^16^. These two ways are correlated with each other, leading to the rapid degradation of Ru active sites. In recent years, research has focused on boosting structural stability of RuO_2_ or Ru-based electrocatalysts via hybridization with IrO_2_^17–24^. However, maintaining sufficient activity is practically difficult because the Ir dopants limit the flexibility of Ru redox^25,26^.

Theoretically, the over-oxidation of Ru site is hindered thermodynamically if the formation energy of oxygen vacancies is greater than that for redox H_2_O/O_2_^27^. For example, it was reported that stability and activity of RuO_2_ nanoparticles can be boosted by reinforcing Ru−O bonding via surface heat treatment^10^. In recent years, foreign element-doping was developed to reinforce Ru−O bonding via enlarging the localized gap between O *2p* band centres and Fermi level^3,28–30^. For example, co-doping of W and Er tuned electronic structure of RuO_2_ via charge redistribution that significantly reduced Ru dissolution and lowered adsorption energies for oxygen intermediates^31^. However, most reported dopants on RuO_2_ have fixed interaction with the Ru site, resulting in limited success in boosting activity or stability^32–35^. Even though conventional heteroatom doping strengthens the lattice oxygen in RuO_2_ at small overpotential via electronic structural redistribution, the stability is not sufficient for practical application because of demetallation of the modified-Ru site at large overpotentials^36^. Therefore, dopants that can tune OER performance via dynamic electron distribution under differing potentials are practically attractive. Reported findings have demonstrated incorporation of high-valence metals, such as W and Ta to stabilize low-charge Ir/Ru^31,37^. Doping with Re, an element with multiple oxidation states between −3 and +7, into RuO_2_, it is possible to build adaptive active sites with dynamic electron transfer. In addition, rhenium oxide is an acidic oxide with strong metal−oxygen bonds. Therefore, Re dopants stabilize the lattice oxygen and low-charge metal active sites concurrently.

Here we report a Re-doped rutile RuO_2_ (Re_0.06_Ru_0.94_O_2_) electrocatalyst that exhibits excellent performance for acidic OER. We show that Re dopants in Re_0.06_Ru_0.94_O_2_ undergo a dynamic electron accepting-donating that adaptively boosts activity and stability, and that this is different from conventional dopants with static dopant electron redistribution. We use operando X-ray absorption spectroscopy (XAS) to confirm that the Re dopants gain electrons from Ru site at the on-site potential to activate OER, and donate electrons back at large overpotential to prevent Ru dissolution. We demonstrate that activity and stability of Re_0.06_Ru_0.94_O_2_ are therefore significantly boosted. We confirm using judiciously combined in situ characterizations and density functional theoretical (DFT) computation that the Re doping reduces adsorption energies for oxygen intermediates, and reinforces the Ru−O bonding in the adsorbate evolution mechanism (AEM) pathway. We conclude that dynamic dopants can be used to boost activity and stability of active sites and therefore guide practical design of adaptive electrocatalysts.

## Results

### Electrocatalytic performance for Re_0.06_Ru_0.94_O_2_

**Fig. 1:**
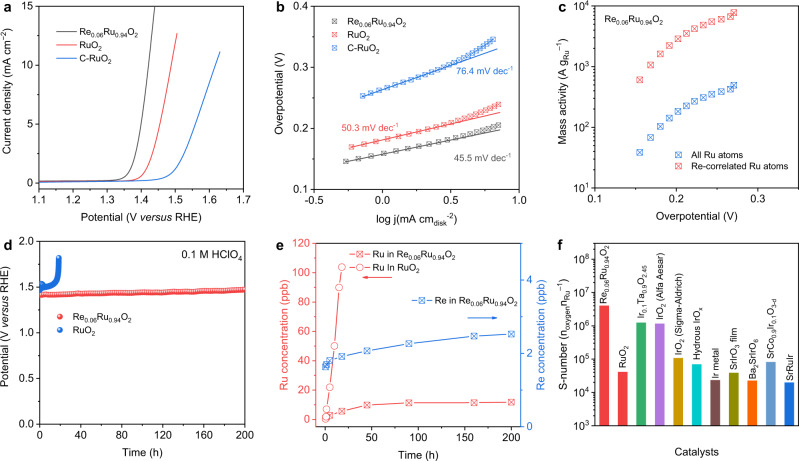
OER performance. **a** LSV curves and **b** Tafel plot for Re_0.06_Ru_0.94_O_2_, RuO_2_, and commercial RuO_2_ in O_2_-saturated 0.1 M HClO_4_ (RHE = reversible hydrogen electrode). **c** Comparison of mass activity for Ru atoms in Re_0.06_Ru_0.94_O_2_ as a function of overpotential. **d** Constant current chronopotentiometric stability measurements at anodic current density 10 mA cm^−2^ for Re_0.06_Ru_0.94_O_2_ and RuO_2_. **e** Dissolved Ru (left ordinate - *y* axis) and Re (right ordinate - *y* axis) ion concentration in electrolyte for Re_0.06_Ru_0.94_O_2_ and RuO_2_ determined via ICP-MS. **f** S-number for Re_0.06_Ru_0.94_O_2_ and RuO_2_ catalyst and Ir-based OER catalysts in acid.

The Re was doped in RuO_2_ via a molten-salt method, and the chemical formula for Re_0.06_Ru_0.94_O_2_ was determined by inductively coupled plasma mass spectrometry (ICP-MS) (Supplementary Fig. 1). The pristine RuO_2_ and Re_0.06_Ru_0.94_O_2_ were analyzed by different characterizations (Supplementary Figs. 2–6). OER performances for Re_0.06_Ru_0.94_O_2_ and other control samples were determined in O_2_-saturated 0.1 M HClO_4_ electrolyte. Figure 1a, b presents the linear sweep voltammetry (LSV) curves and corresponding Tafel slopes for Re_0.06_Ru_0.94_O_2_, RuO_2_, and commercial RuO_2_ (denoted C-RuO_2_). Re_0.06_Ru_0.94_O_2_ exhibited an overpotential of 190 mV at current density 10 mA cm^−2^ (*η*_10_) with a Tafel slope 45.5 mV dec^−1^, outperforming RuO_2_ with 258 mV and 50.3 mV dec^−1^ and, C-RuO_2_, 388 mV and 76.4 mV dec^−1^, respectively^38^. LSV curves without i-R compensation are presented in Supplementary Fig. 7a as a reference. The mass activity for Re_0.06_Ru_0.94_O_2_ is 500 A g^−1^ at overpotential 272 mV, which is greater than RuO_2_ of 156 A g^−1^ at 272 mV and, C-RuO_2_ of 18 A g^−1^ at 290 mV, respectively (Supplementary Fig. 7b). In addition, Re-correlated Ru sites (Ru active sites linked to Re atoms) exhibit a mass activity of 7811 A g^−1^ (Fig. 1c), which is greater than most acidic OER catalysts. The turnover frequency (TOF)^39^ for Re_0.06_Ru_0.94_O_2_ at overpotential 272 mV is 0.17 s^−1^ (Supplementary Fig. 8a), which is an order of magnitude greater than for C-RuO_2_ of 0.004 s^−1^. Because recorded current might be affected by the Re and Ru reconstruction, we measured the Faradaic efficiency (FE) for OER by real-time monitoring O_2_. The Re_0.06_Ru_0.94_O_2_ exhibited a FE of ~100% at current density from 5 to 25 mA cm^−2^, confirming high OER selectivity (Supplementary Fig. 8b). A comparative performance of Re_0.06_Ru_0.94_O_2_ with reported OER electrocatalysts is presented in Supplementary Table 1. It is seen from the table that Re_0.06_Ru_0.94_O_2_ exhibits comparatively excellent OER performance in acidic electrolytes, better than other recently reported noble metal-based electrocatalysts. The doping impact of Re on OER performance was determined (Supplementary Figs. 9–14) via a series of Re-RuO_2_ with different Re. It was found that Re doping does not measurably change rutile structure for Re-RuO_2_.

Durability of Re_0.06_Ru_0.94_O_2_ catalyst is an important parameter for acidic OER electrocatalysts^40,41^. Figure 1d presents the chronopotentiometry data for Re_0.06_Ru_0.94_O_2_ at constant current density 10 mA cm^−2^ via loading on carbon paper with a loading mass 0.2 mg_cata_ cm^−2^. It is seen that the potential for Re_0.06_Ru_0.94_O_2_ remained steady (constant) for a continuous 200 h test. However, RuO_2_ exhibited a rapid activity decay within 19 h, likely the result of Ru dissolution in the acidic electrolyte. Importantly, carbon paper is not an ideal support for acidic OER durability testing because of due substrate passivation^42–46^. As a result, chronopotentiometry is not always a reliable technique to determine stability of acidic OER catalysts on carbon paper. Detecting catalyst mass losses during OER can provide quantitative information that distinguishes between different degradation mechanisms^42^. Therefore, Ru dissolution in different catalysts was determined to confirm a degradation mechanism. Figure 1e and Supplementary Table 2 present the time-dependent Ru and Re concentration in the electrolyte, corresponding to Fig. 1d. Dissolved Ru concentration for RuO_2_ increased rapidly to 104 ppb within 20 h, equaling 2.7% Ru loss. In contrast, dissolved Ru and Re for Re_0.06_Ru_0.94_O_2_ were significantly less at 11.8 and 2.5 ppb after stability testing for 200 h. By converting to mass loss of the metal specie, there was just 0.34% of loss with Ru, and 0.62% with Re. In addition, Ru and Re dissolution rates in Re_0.06_Ru_0.94_O_2_ decreased over time, evidencing a stable structure during OER. The stability number (S-number) for the catalysts was determined via measuring oxygen produced and dissolved metal ion concentration in the electrolyte (Supplementary Fig. 15)^40^. Both RuO_2_ and Re_0.06_Ru_0.94_O_2_ show a time-dependent S-number, similar to the recently reported work^47^. The S-number of the catalysts increases with time, and is attributed to the slower Ru dissolution rate. Significantly, the S-number for Re_0.06_Ru_0.94_O_2_ is significantly greater than that reported for Ru-based electrocatalysts and, importantly, comparable with Ir-based catalysts (Fig. 1f)^28,37,40^.

### Structural evolution of Re_0.06_Ru_0.94_O_2_

**Fig. 2:**
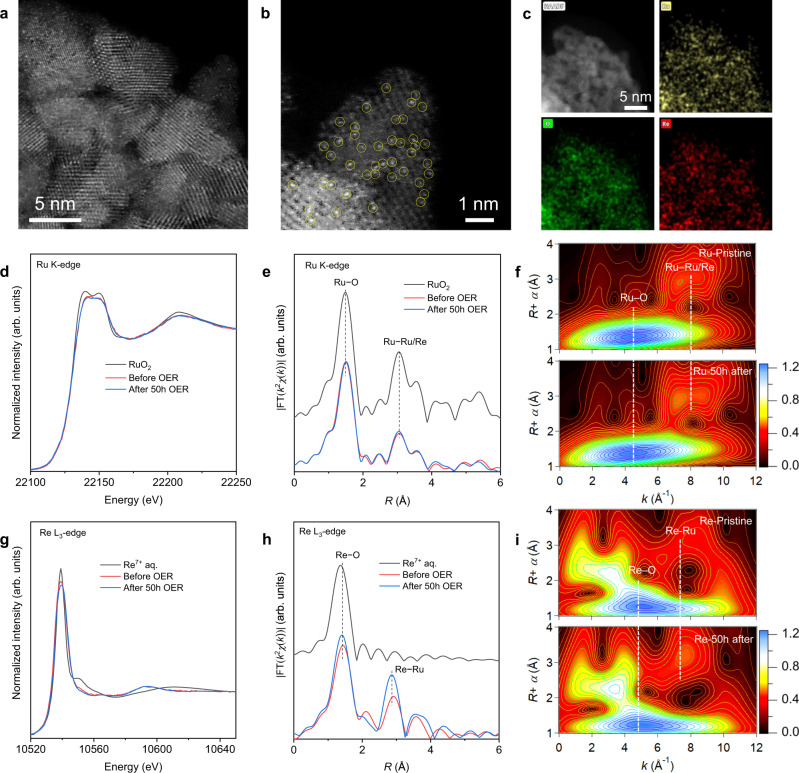
Structural characterization of Re_0.06_Ru_0.94_O_2_ after 50 h OER in acid. **a**, **b** Aberration-corrected HAADF-STEM image of Re_0.06_Ru_0.94_O_2_ after 50 h OER. Re sites are labeled with yellow-color circles. **c** EDS mapping images of Re_0.06_Ru_0.94_O_2_ catalyst after 50 h OER. **d**, **e** Ru K-edge XANES spectra and FT *k*^2^-weighted EXAFS signals for RuO_2_, Re_0.06_Ru_0.94_O_2_ before and after 50 h OER. **f** Comparison of Ru K-edge WT-EXAFS for RuO_2_, Re_0.06_Ru_0.94_O_2_ before and after 50 h OER. **g**, **h** Re L_3_-edge XANES spectra and FT *k*^2^-weighted EXAFS signals for Re^7+^ in aqueous solution, Re_0.06_Ru_0.94_O_2_ before and after 50 h OER. **i** Comparison of Re L_3_-edge WT-EXAFS for Re_0.06_Ru_0.94_O_2_ before and after 50 h OER.

The stability of Re_0.06_Ru_0.94_O_2_ was determined via crystal structure and chemical states of samples after 50 h chronopotentiometric test in 0.1 M HClO_4_. Re single atoms are anchored in the RuO_2_ crystal without aggregation or reconstruction after OER, as confirmed in the aberration-corrected high-angle annular dark-field scanning transmission electron microscopy (HAADF-TEM) images in Fig. 2a, b, evidencing the stability of Re dopants in the rutile structure during catalyzing. Corresponding elemental mapping confirmed that Re atoms are distributed uniformly in Re_0.06_Ru_0.94_O_2_ nanoparticles, similar to that for the pristine samples (Fig. 2c). Significantly, RuO_2_ also exhibited a rutile structure after 20 h OER testing (Supplementary Fig. 16), evidencing that degradation of active site does not change crystal structure of the matrix meaningfully. X-ray photoelectron spectroscopy (XPS) and synchrotron-based near-edge X-ray absorption fine structure (NEXAFS) spectroscopy were used to determine the electronic state for Re_0.06_Ru_0.94_O_2_ and RuO_2_ prior to and after OER testing. As is presented in Supplementary Figs. 5 and 17a, the valence state for Ru in RuO_2_ after OER increased when compared with that for pristine RuO_2_, whereas Ru sites in Re_0.06_Ru_0.94_O_2_ were more stable than in RuO_2_. In addition, the Re sites in Re_0.06_Ru_0.94_O_2_ were highly stable during OER (Supplementary Fig. 17b). O-related spectra for RuO_2_ evidence higher average Ru valence states and charge redistribution compared with pristine sample, that is caused by Ru dissolution-induced catalyst degradation (Supplementary Figs. 17c, d)^48,49^.

Ru K-edge and Re L_3_-edge XAS prior to and after 50 h OER were characterized to determine the local coordination environment-change of Re_0.06_Ru_0.94_O_2_ after stability testing. Compared with pristine Re_0.06_Ru_0.94_O_2_, the Ru K-edge X-ray absorption near-edge spectroscopy (XANES) of the sample after OER exhibited an (apparently) unchanged valence state with dominated Ru^4+^ (Fig. 2d), consistent with XPS results. Besides, Fourier-transformed magnitudes of the Ru K-edge extended X-ray absorption fine structure (EXAFS), which exhibited two main peaks at ~1.5 and 3.1 Å, showed slight change in Re_0.06_Ru_0.94_O_2_ (Fig. 2e). They corresponded to the nearest-neighbor Ru−O and next nearest-neighbor Ru−Ru/Re coordination shells, respectively, as confirmed by EXAFS wavelet transformed (WT) analysis which reveals two intensity maxima at ~4.2 and 8.0 Å^−1^ (Fig. 2f). Further EXAFS fitting showed that the Ru−O peak can be divided into two distinct sub-shells with interatomic distances of 1.92 Å (coordination number *N* is 1.8) for Ru−O1 and 2.01 Å (coordination number *N* is 4.1) for Ru−O2, similar to the pristine sample (Supplementary Fig. 18 and Supplementary Table 3). In addition, the apparently unchanged coordination number excludes the formation of highly concentrated Ru/Re vacancies. Therefore the coordination environment for Ru in Re_0.06_Ru_0.94_O_2_ following OER is confirmed to be unchanged, evidencing that Re doping strengthens the Ru−O bond and prevents Ru dissolution. In addition, Re L_3_-edge XANES confirms that Re_0.06_Ru_0.94_O_2_ after OER exhibits a similar Re valence state (6.33) to the pristine sample (6.38) (Fig. 2g and Supplementary Fig. 19). The Re L_3_-edge FT-EXAFS spectrum and WT-EXAFS for Re_0.06_Ru_0.94_O_2_ prior to and after OER demonstrate a rather similar profile to the Ru K-edge (Fig. 2h, i), confirming that the Re sites are stable in the RuO_2_ matrix without formation of ReO_x_-related species (Supplementary Fig. 20 and Supplementary Table 4). It should be noted that the FT-EXAFS of Ru K-edge and Re L_3_-edge shows similar shape, confirming the substitutional doping of Re atoms in RuO_2_.

### Dynamic electron transferring in Re_0.06_Ru_0.94_O_2_

**Fig. 3:**
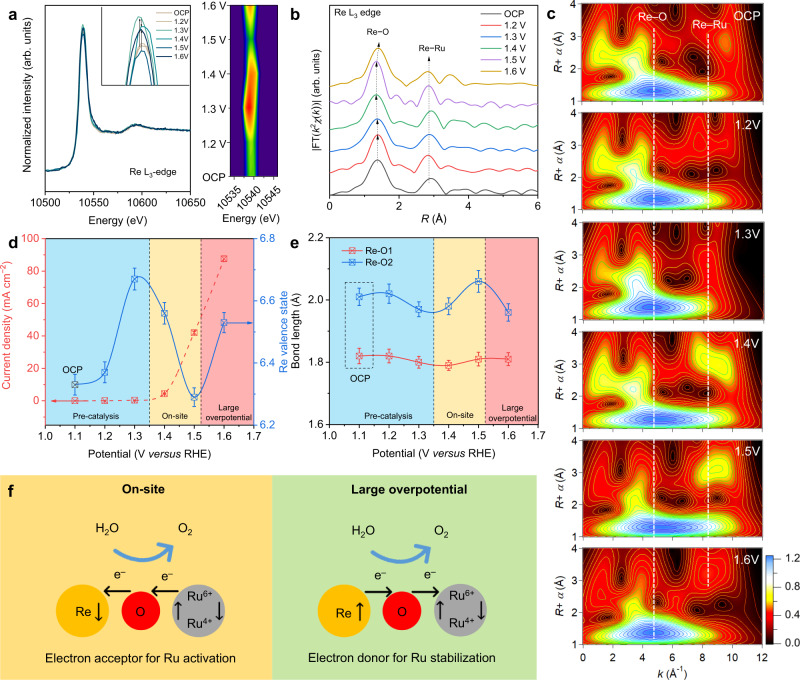
Operando XAS characterization of Re_0.06_Ru_0.94_O_2_ confirming dynamic process. **a** Re L_3_-edge XANES spectra for Re_0.06_Ru_0.94_O_2_ at differing potentials in O_2_-saturated 0.1 M HClO_4_. Right part: contour plot of the Re L_3_-edge white peak intensity. **b** FT-EXAFS signals for Re_0.06_Ru_0.94_O_2_ corresponding to (**a**). **c** Comparison of Re L_3_-edge WT-EXAFS plots for Re_0.06_Ru_0.94_O_2_ from OCP to 1.6 V. **d** Change in Re valence state and OER current as a function of applied potential. **e** Change in bond length for Re–O1 and Re–O2 coordination shells. The operando XAS measurements were repeated three times to produce the error bar. **f** Schematic for dynamic electron transfer in Re_0.06_Ru_0.94_O_2_. In OER on-site region, Re dopants gain electrons from neighboring Ru to boost OER. At large overpotential, Re dopants donate electrons back to Ru and prevent dissolution of active sites.

Because post-reaction characterizations cannot be used to determine the change in materials during OER, operando Re L_3_-edge XAS at different overpotentials were carried out to understand the interaction between Re and sites in situ. Changes in the local electronic and atomic structures from open-circuit potential (OCP) to 1.6 V were determined via XANES and EXAFS analysis. As is shown in Fig. 3a–c, the Re dopants in Re_0.06_Ru_0.94_O_2_ exhibit dynamic electron accepting-donating, which, importantly, is different from conventional dopants. Prior to OER, the valence state of Re increased because of Re oxidation with the increase of oxidizing potential. At overpotentials around OER on-site, the Re valence decreased significantly to less than the pristine state. However, at large overpotential, the valence state of Re again increased. This dynamic electron transfer only occurs with applied potential, and is not observed in the post-reaction XAS measurements, Fig. 2. The operando XAS measurement was repeated three times to confirm this unique dynamic electron transfer. Based on the spectral evolution for Re L_3_-edge, the OER on Re_0.06_Ru_0.94_O_2_ was divided into three stages, (1) pre-catalytic from OCP to 1.35 V, (2) on-site catalytic stage from 1.35 to 1.5 V and, (3) large-overpotential stage from 1.5  to 1.6 V. To determine reaction mechanism in situ the change of Re valence state (Fig. 3d) and the bond length for Re–O1 and Re–O2 (Fig. 3e, Supplementary Fig. 21, and Supplementary Table 4) were analyzed.

In Stage (1), the average valence state for Re of Re_0.06_Ru_0.94_O_2_ increased from 6.33 to 6.67 when compared with the pristine sample (Fig. 3a, d), while a similar intensity increase was apparent in the first peaks in FT-EXAFS spectra (red-color region, Fig. 3b). In particular, with applied potential increased from OCP to 1.3 V, the Re–O peak shifted positively from 1.36  to 1.38 Å. To determine the reason for these changes, WT analysis of EXAFS spectra was conducted, which provides R- and k-space information and discriminates the backscattering atoms (Fig. 3c). For the contour intensity maximum corresponding to the FT-EXAFS peak for Re–O at 1.36 Å, an evident intensity increase is observed at 9.0 Å^−1^ (denoted by a dashed line in Fig. 3c), which agrees well with the location of Re–Ru scattering. These changes confirm that this stage is pre-catalytic, in which the increased valence state of Re is due to the oxidizing potential, and not OER reaction.

With an applied potential >1.3 V, Stage (2), the most apparent feature is that the Re valence state decreased significantly from 6.67 to 6.29, as is shown in Fig. 3d. In addition, the bond length for Re–O2 elongates obviously with the increase of applied potentials, evidencing a dynamic electron-transfer amongst Ru, Re and adsorbed oxygen intermediates (Fig. 3e), demonstrating that Re dopants gain electrons from Ru site to tune electronic structure, and activate Ru sites. It is widely acknowledged that the high-valence Ru species are the active sites for OER. Therefore, the Re gains electrons from Ru at Stage (2), to facilitate the formation of high-valence Ru sites to boost OER. This stage also explains why the activity of Re_0.06_Ru_0.94_O_2_ is greater than pure RuO_2_.

In Stage (3), with the applied potential reaching 1.5 V, the Re valence state increases again from 6.29 to 6.53 (Fig. 3d), evidencing that the Re dopants donate the electrons back to Ru active site. Importantly, WT-EXAFS analysis highlights the appearance of the Ru–Re scattering signal at 1.3 V, and its disappearance at 1.6 V, to demonstrate a dynamic bonding length change between Re–Ru coordination shell, which is associated with a contraction of Re−O2 bond length. This stage evidences that the Re dopants protect the active site from dissolution at large overpotential by donating the electrons back to Ru to maintain stable catalyzing.

We investigated the Ru sites via operando Ru K-edge XAS to determine the change for Ru cations. In contrast to dynamic change for Re cations with applied potential, it was found that both operando Ru K-edge XANES and EXAFS exhibit only ‘slight’ change during catalysis (Supplementary Fig. 22), as was confirmed by detailed Ru K-edge EXAFS fitted analyses (Supplementary Figs. 23–24 and Supplementary Table 3). It is widely acknowledged that XAS analyses reflect average information for all Ru atoms in Re_0.06_Ru_0.94_O_2_. The XAS signal for Ru−O–Ru site dominates and overlaps that for Ru−O–Re site because of the low doping amount of Re in Re_0.06_Ru_0.94_O_2_, leading to the unchanged Ru K-edge spectra. Therefore, we focused mainly on the Re L_3_-edge to determine the dynamic behavior of Ru−O–Re sites.

Operando XAS results confirm that the electron transfer between Re and Ru site is potential-depended in which the function for Re varies. As is summarized in Fig. 3f, the Re_0.06_Ru_0.94_O_2_ electrocatalyst undergoes a three-step dynamic electron transfer during OER, which is different from conventional RuO_2_ (Supplementary Fig. 25). At the pre-catalysis stage, the increase in Re valence state is due to the gradually increased oxidizing potential. During OER, Stage (2), the Re atoms gain electrons from Ru site via the O bridge to boost Ru sites for catalyzing, which explains the boosted activity of Re_0.06_Ru_0.94_O_2_. At large overpotentials, Stage (3), the Re dopants donate electrons back to prevent over-oxidation of Ru active sites and formation of H_2_RuO_5_ species. Therefore, the stability of Re_0.06_Ru_0.94_O_2_ is significantly boosted to greater than most Ir-based electrocatalysts. Consequently, it is concluded that the Re dopants act as a dynamic electron reservoir that achieves an adaptive tune of the OER active site in situ.

### Mechanistic analysis of dynamic electron transfer

The boosted stability of most acidic OER electrocatalysts is from the change in reaction pathway from LOM to AEM^28,31,50^. This is because the AEM pathway involves multiple intermediates of *OH, *O, and *OOH without lattice oxygen participation, leading to long-term catalyzing^51^. Therefore, additional in situ measurements were conducted to determine the impact of dynamic electron transfer on the reaction mechanism for Re_0.06_Ru_0.94_O_2_. It is widely acknowledged that conventional RuO_2_ catalyzes OER in a LOM-involved pathway (Supplementary Fig. 26)^16,52^ in which the mobilized crystal structure is caused by the oxygen vacancies and leached Ru site. However, the Re_0.06_Ru_0.94_O_2_ exhibited significantly less Ru dissolution than RuO_2_, most probably because of the changed reaction pathway from LOM to AEM.

**Fig. 4:**
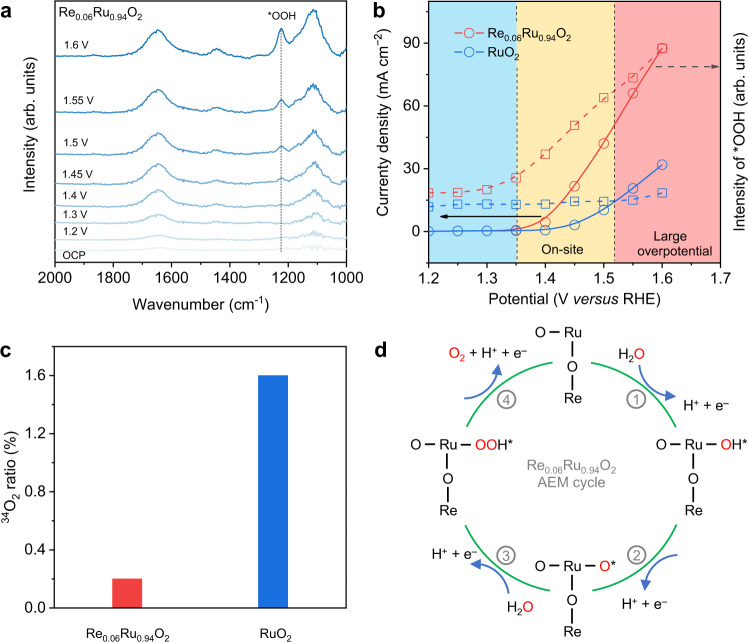
Reaction mechanism on Re_0.06_Ru_0.94_O_2_ and RuO_2_. **a** In situ ATR-SEIRAS spectra for Re_0.06_Ru_0.94_O_2_ during multi-potential steps. **b** Potential dependence of band intensity of characteristic vibration adsorption of surface-adsorbed *OOH. **c** ^34^O_2_ ratio determined via normalization of DEMS signal for Re_0.06_Ru_0.94_O_2_ and RuO_2_ in 0.05 M H_2_SO_4_-H_2_^16^O. **d** Illustration for AEM pathway on Re_0.06_Ru_0.94_O_2_ toward acidic OER.

We used in situ attenuated total reflectance surface-enhanced infrared absorption spectroscopy (ATR-SEIRAS) to confirm the OER mechanism on Re_0.06_Ru_0.94_O_2_. This is because this technique can examine the potential-dependent surface reaction intermediates. The in situ ATR-SEIRAS spectra for Re_0.06_Ru_0.94_O_2_ at differing working potential (Fig. 4a) exhibit a distinct absorption peak at 1224 cm^−1^, which is attributed to the O–O stretching of surface-adsorbed *OOH, a typical intermediate for AEM pathway^53^. Peak intensity increased linearly from OER on-site to 1.6 V, evidencing a constant AEM pathway at different catalyzing stages (Fig. 4b). In comparison, RuO_2_ exhibits unidentifiable *OOH with weaker intensity after OER on-site (Fig. 4b and Supplementary Fig. 27), confirming a LOM-involved pathway. Noted that an identifiable *OOH peak appeared at an applied potential on RuO_2_ of 1.6 V (Supplementary Fig. 28), evidencing that a combined LOM-AEM pathway dominates the reaction at large overpotential. In addition, the LSV curves for Re_0.06_Ru_0.94_O_2_ and RuO_2_ (Supplementary Fig. 29) were carefully analyzed. The RuO_2_ exhibited an apparent activation-deactivation that agreed well with features of the LOM pathway. In contrast, Re_0.06_Ru_0.94_O_2_ exhibited stable activity under cycling, evidencing a stable AEM characteristic.

To further confirm the AEM reaction pathway on Re_0.06_Ru_0.94_O_2_ and RuO_2_, ^18^O isotope-assisted operando differential electrochemical mass spectrometry (DEMS) analyzes were carried out, which can directly differentiate AEM and LOM. Samples were first labeled using H_2_^18^O- contained electrolytes (Supplementary Figs. 30–31). In a typical LOM pathway, the lattice oxygen labeled with ^18^O_ads_ couples with ^16^O in the electrolyte to generate ^34^O_2_. In contrast, the AEM pathway produces ^36^O_2_ from water-splitting because no lattice oxygen participates in OER^28^. As is shown in Fig. 4c and Supplementary Fig. 32, RuO_2_ produces a clear ^34^O_2_ signal with high intensity, significantly greater than that for Re_0.06_Ru_0.94_O_2_. Given that both catalysts exhibit a similar ECSA (Supplementary Fig. 10), the influence of physically adsorbed H_2_^18^O is excluded. Quantitatively, the RuO_2_ produces 1.6% ^34^O_2_, evidencing a LOM-contained pathway. However, the Re_0.06_Ru_0.94_O_2_ produces just 0.3% ^34^O_2_, close to the ^18^O content in natural water and air, evidencing the AEM pathway (Fig. 4d). Importantly, it was reported that <0.2% of evolved oxygen contains an oxygen atom originating from RuO_x_^11^. The difference with our findings is because of poor crystallinity of our sample obtained from the molten salt method, which has more active lattice oxygen. It is concluded that the in situ FTIR, online DEMS and post-reaction XPS measurements confirm the reaction pathway on Re_0.06_Ru_0.94_O_2_ and RuO_2_. The dynamic Re dopants change the reaction pathway on Re_0.06_Ru_0.94_O_2_ from LOM to AEM, leading to boosted OER.

### Computations for activity and stability origin

**Fig. 5:**
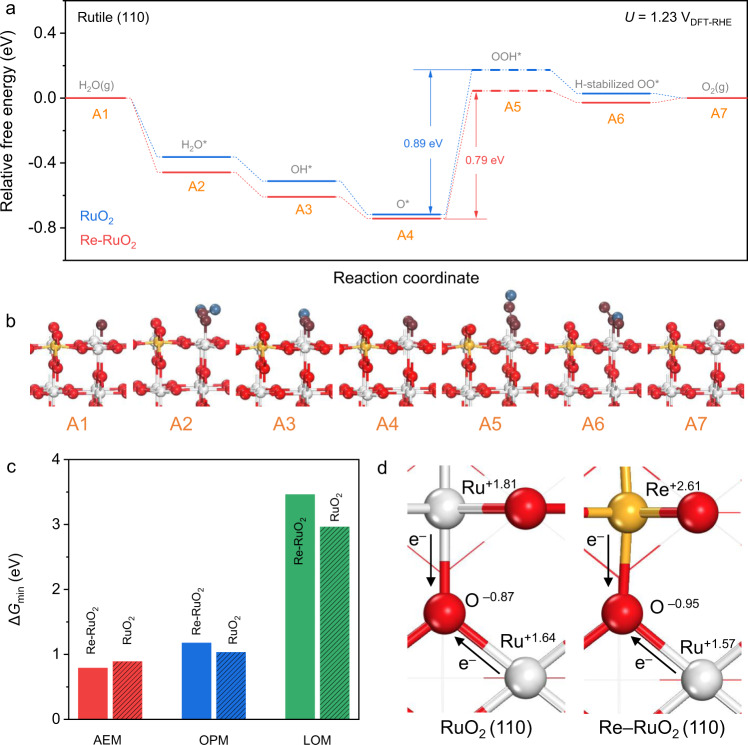
DFT simulation. **a**, **b** Free energy diagram for OER on unsaturated Ru site of Re_0.06_Ru_0.94_O_2_ and RuO_2_ at 1.23 V *versus* RHE, showing six (6) possible intermediates for the (110) surfaces. Dashed lines indicate unstable –OOH precursor states, shown as H-stabilized OO*. **c** Minimum activation energy for different reaction pathways for Re_0.06_Ru_0.94_O_2_ and RuO_2_. **d** Oxidation state for RuO_2_ and Re_0.06_Ru_0.94_O_2_ described by differences in electron transfer based on Bader charge computations. Black-color arrows show direction of electron transfer.

DFT computations were performed to provide qualitative analyses of the OER mechanism and the stability origin of Re_0.06_Ru_0.94_O_2_. Based on the STEM and X-ray powder diffraction (XRD) findings, a Re-doped rutile RuO_2_ (110) slab model was constructed. Various doping sites were tested, and the most stable structure was selected. Figure 5a presents the OER with AEM pathway on RuO_2_ and Re-RuO_2_ (110), based on in situ measurements. The unsaturated Re site on Re-RuO_2_ was inactive for OER because of the too strong adsorption of key oxygen intermediates (Supplementary Fig. 33). Reaction intermediate configurations on Re-RuO_2_ (110) are illustrated in Fig. 5b (Those for RuO_2_ are shown in Supplementary Fig. 34). The initial step is the formation of a deoxygenated surface (A1), followed by adsorption of a water molecule on the Ru site (A2) together with subsequent formation of OH* (A3) and O* (A4) species from H_2_O* deprotonation. Then, the adsorption of another water molecule occurs to compose an OOH* (A5). It should be noted that this step is unstable, in which the OOH* donates the proton to the neighboring oxygen to form an H-stabilized OO* species (A6). Molecular oxygen is then formed from this H-stabilized OO* (A7)^52^. As is shown in Fig. 5a, the step from A4 to A5 is the most energetically difficult step under 1.23 V_DFT-RHE_ (defined by a computational hydrogen electrode model)^54^, where Re-RuO_2_ exhibits reaction energy of 0.79 eV, 0.1 eV less than that on RuO_2_. Thermodynamic advantage of Re-doped RuO_2_ over pure RuO_2_ is therefore demonstrated. In addition to LOM and AEM pathways, a new oxide path mechanism (OPM) pathway has been confirmed that allows direct O–O radical coupling without generation of oxygen vacancy defects and extra reaction intermediates (Supplementary Fig. 35)^55,56^. Therefore, Fig. 5c compares the minimum required energy to activate AEM, LOM, and OPM pathways on, respectively, Re_0.06_Ru_0.94_O_2_ and RuO_2_. The AEM pathway is seen in the figure to be the most energetically favorable compared with the other two on both surfaces, evidencing that it dominates OER under the equilibrium potential. Qualitative analyses of OER mechanism for RuO_2_ and Re-RuO_2_ with metal vacancies were assessed via DFT computation. Importantly, the structure for Re-RuO_2_ with a Re vacancy is the same as for RuO_2_ with a Ru vacancy following stabilization. We considered Re (vac-RuO_2_) and Ru (vac-Re-RuO_2_) vacancies therefore on Re-RuO_2_ to determine the impact on electrocatalytic performance. As is seen in Supplementary Figs. 36 and 37, although the sample with metal defects exhibits optimized OPM thermodynamic energy, it is less than that for AEM on Re-RuO_2_, confirming the advantage of Re-RuO_2_ over RuO_2_ or metal defects.

Although the in situ measurements appear to evidence that OER on RuO_2_ is likely to occur through a combined LOM-AEM pathway, we hypothesize that the LOM pathway on RuO_2_ is activated because of the applied potential and corrosive acidic environment that mobilizes the Ru–O bond and leads to the generation of O vacancies. This greater tendency for the AEM pathway with Re-RuO_2_ and stability origin was examined via surface oxidation states. As is seen from Bader charge analysis (Fig. 5d), more negative charge is transferred from Re to Ru via the O bridge on the Re-RuO_2_ surface compared with RuO_2_, confirming that Re doping reinforces the stability of Ru–O bond, and promotes the AEM under acidic media. An acknowledged drawback, however, is that DFT computations cannot simulate the dynamic electron transfer because the bond length and electronic structure differ with applied potential change. However, the DFT does provide a reasoned, qualitative evaluation of Re_0.06_Ru_0.94_O_2_ that is important to establish the activity and stability origin of the catalysts.

## Discussion

In summary, we report a Re_0.06_Ru_0.94_O_2_ catalyst with dynamic Re dopants that exhibits significantly boosted activity and stability for acidic OER. Interaction between aciduric Re dopant and Ru active site is tuned by dynamic electron-accepting-donating in which the Re gains electrons to activate the Ru site at on-site overpotential and donates electrons back at large overpotential to prevent Re dissolution. Consequently, Re_0.06_Ru_0.94_O_2_ exhibits intrinsic activity and corrosion resistance to make amongst the best acidic OER electrocatalysts. Based on combined, judicious in situ measurement and DFT computation, we confirm that boosted catalytic activity and corrosion resistance originates from modified electronic structure via dynamic electron transfer, facilitating the change of pathway on Re_0.06_Ru_0.94_O_2_ from LOM to AEM and lessens adsorption energies for oxygen intermediates in the active site. We conclude that dynamic dopants can be used to boost the activity and stability of active sites and therefore guide the design of adaptive electrocatalysts for improved clean energy conversions.

## Methods

### Chemicals

Sodium nitrate (≥99%), ruthenium (III) chloride hydrate (ruthenium content, 40.00 to 49.00%), and ruthenium (IV) oxide (≥99.9%) were purchased from Sigma-Aldrich without further purification. Sodium perrhenate (99%) was purchased from Alfa Aesar, Australia. ^18^O Water (^18^O, 97%+) was purchased from Novachem, Australia.

### Catalyst synthesis

Re_0.06_Ru_0.94_O_2_ were synthesized via a molten-salt method. Typically, 5 g NaNO_3_ and 100 mg NaReO_4_ were mixed in a 50 mL porcelain crucible. The crucible was transferred to a muffle furnace and heated to 360 °C for 10 min. 40 mg RuCl_3_·xH_2_O power was added to the crucible (with lid) and held for 5 min. NO_2_ brown-color gas was generated when adding RuCl_3_·xH_2_O because of oxidation of Ru^3+^. The crucible was removed from the furnace and cooled to room temperature (25 °C) under ambient conditions. The product was washed with deionized water to remove salt and obtained via vacuum filtration (Millipore 0.22 µm). RuO_2_ was synthesized without addition of NaReO_4_. Re_0.03_Ru_0.94_O_2_, Re_0.04_Ru_0.94_O_2_, Re_0.05_Ru_0.94_O_2_, Re_0.06_Ru_0.94_O_2_, and Re_0.1_Ru_0.9_O_2_ were prepared via varying NaReO_4_ to, respectively, 20, 40, 80, and 150 mg.

### Electrochemical measurement

Typically, 2 mg of catalyst was dispersed in 950 μL of deionized water. 50 μL 5 wt% of Nafion/water was added to the catalyst dispersion. 10 µL of the catalyst dispersion (2 mg mL^–1^) was dropped onto a 5 mm glassy-carbon rotating disk electrode (0.1 mg_cata_ cm^−2^) serving as the working electrode. The reference electrode was Ag/AgCl in 3.5 M AgCl–KCl solution, and the counter electrode was a graphite-rod. The reference electrode was calibrated in hydrogen-saturated 0.01 M HClO_4_. The calibrated value for Ag/AgCl in 3.5 M AgCl–KCl was equal to 0.214 (close to the theoretical value of 0.205). To evaluate OER for different catalysts, the working electrode was put through 10 cyclic voltammetry (CV) scans between 1.1 to 1.6 V at a 20 mV s^−1^ to clean and stabilize the surface of the catalyst. All potentials were referenced to RHE via adding (0.214 + 0.059 × pH) V, and all polarization curves were corrected for iR compensation within the cell. A flow of O_2_ was maintained over the electrolyte during experiment. The working electrode was rotated at 1600 rpm to remove O_2_ gas formed on the catalyst surface. Electrochemical data were corrected for uncompensated series resistance R_s_ that was determined via open-circuit voltage. A 90% i-R compensation was selected to determine R_s_. The final polarization curve was determined via i-R compensation. To determine metal dissolution in the electrolyte, a stability test was conducted in an H-cell. The stability measurements were carried out by air-brush spraying the catalysts uniformly on the carbon with a loading mass of 0.2 mg_cata_ cm^−2^. The stability test was conducted under 25 °C. The cathode and the anode chamber were separated by a Nafion film to obviate re-deposition of Ru/Re on the counter electrode. Carbon paper (effective area 1 cm^−2^) was used as working electrode with a loading mass of 0.2 mg_cata_ cm^−2^.

### In situ ATR-SEIRAS measurement

In situ ATR-SEIRAS was carried out with a Nicolet iS20 spectrometer with an HgCdTe (MCT) detector cooled with liquid N_2_ and a VeeMax III (PIKE Technologies) accessory. Electrochemical tests were conducted in a custom-made, three-electrode electrochemical single-cell. A Pt-wire and a saturated Ag/AgCl were used as, counter and reference electrodes. A fixed-angle, Au-coated Si prism (60°) was used to load catalysts and served as the working electrode. Au thin-layer with a thickness 40 nm was coated by magnetron sputtering. The reflecting plane of the Si prism was polished with diamond compound (0.05 µm, Kemet. Int. Ltd.), and sonicated in acetone, ethanol, and water before sputtering. For ATR-SEIRAS measurement, 32 scans were collected with a spectral resolution of 4 cm^–1^ for each spectrum. The background spectrum of the working electrode was recorded in an open-circuit condition. The recorded spectra were processed via OMNIC software.

### Online DEMS with isotope labeling

The online DEMS system used was HPR-40, HIDEN. Measurements were determined using a modified DEMS cell (cell A) at atmospheric pressure. The pressure difference between the cell and the vacuum chamber allows oxygen species to be drawn into the vacuum chamber for mass spectrometer analysis. The working electrode is a Au-film with 50 nm thickness that is sputtered on a hydrophobic polytetrafluoroethylene membrane with a pore size 0.02 μm (Hangzhou Cobetter Filtration Equipment Co.). Catalyst ink was dropped into the Au-film, and dried. For isotope labeling, 3 mL of 0.05 M H_2_SO_4_ was prepared using H_2_^18^O (97%+) as solvent. Concentrated H_2_SO_4_ of 98%+ was used and not 70% HClO_4_ to reduce any contamination of H_2_^16^O in the solute. Re_0.06_Ru_0.94_O_2_ and the RuO_2_ were subjected to four (4) CV cycles in the potential range, respectively, 1.06 to 1.38 V and 1.1 to 1.42 V *versus* RHE at a scan rate 5 mV s^−1^ for labeling catalyst surface with ^18^O, while mass signals for gaseous products ^32^O_2_, ^34^O_2_, and ^36^O_2_ were recorded. The catalysts were washed with abundant water (H_2_^16^O) and dried in a vacuum-oven for 1 h to remove H_2_^18^O molecules physically attached to the catalyst-layer, the ^18^O-containing species chemically bonded on the surface, remained. Catalysts with isotope-labeled surfaces were operated in a standard electrolyte 0.5 M H_2_SO_4_ with H_2_^16^O as solvent. The mass spectrometer was used to monitor gaseous products, including ^32^O_2_, ^34^O_2_, and ^36^O_2_. Prior to electrochemical measurement, all the electrolytes were purged with high-purity Ar to remove dissolved oxygen.

### Ex situ and operando XAS characterizations

Re L_3_-edge and Ru K-edge XAS data were collected at beamlines 1W1B in the Beijing Synchrotron Radiation Facility (BSRF, operated at 2.5 GeV with a maximum current 250 mA) with a Si (111) double-crystal monochromator^57^. Ex situ XAS measurements were conducted in transmission mode at 25 °C. We used standard N_2_-filled ion chambers to monitor the intensity of the incident and transmitted X-rays. Operando XAS measurements were carried out in the fluorescence model using a homemade in situ cell and an electrochemical workstation. The working electrode is Re_0.06_Ru_0.94_O_2_ loaded on carbon paper. In addition, Pt-wire was used as the counter electrode and Ag/AgCl electrode as the reference electrode. O_2_-saturated 0.1 M HClO_4_ solution was used as the electrolyte. The operando XAS measurements were repeated three times to confirm the trend and build an error bar. We used ATHENA and ARTEMIS modules implemented in IFEFFIT software to analyze XAS data^58^. We conducted background subtraction and normalization to obtain EXAFS data and applied a Hanning window to obtain *χ*(**k**) data Fourier-transformed to real (*R*) space. Least-squares curve-fitting of EXAFS *χ*(**k**) data was carried out in R-space to determine quantitative structural parameters around central atoms. The structural model of XAS was built based on the crystal structures for RuO_2_ and Re-RuO_2_, with scattering amplitudes, phase shifts, and photoelectron mean free path for all paths computed with ab initio code FEFF 8.6.

### Material characterization

X-ray powder diffraction (XRD) data were collected on a Rigaku MiniFlex 600 X-Ray diffractometer. Field-emission SEM imaging was determined on a FEI QUANTA 450 electron microscope. High-angle annular dark-field imaging, and EDS mapping, were determined on a FEI Titan Themis 80-200, operating at 200 kV.

The atomic ratio for Re over Ru was analyzed via ICP-MS, Agilent 7500cx instrument. The catalyst was dispersed and dissolved in aqua regia at temperature 25 °C prior to ICP-MS analysis. Dissolved Ru and Re in the electrolyte were analyzed via ICP-MS by diluting the electrolyte two times prior to measurement.

Synchrotron-based NEXAFS measurements were determined on the soft X-ray spectroscopy beamline at the Australian Synchrotron, which is equipped with a hemispherical electron analyzer and a microchannel plate detector to permit concurrent recording of the total electron, and partial electron, yield. Calibration of XPS data were normalized to the photoelectron current of the photon beam, measured on an Au-grid. Raw XANES data were normalized using Igor Pro 8 software.

### Computational details

All the structures were optimized by density functional theory (DFT) with a periodic plane-wave implementation using Vienna ab initio Package (VASP) code^57,59,60^. Perdew–Burke–Ernzerhof (PBE) functional within the generalized gradient approximation (GGA) was used to model the exchange-correlation energy^61^. The ionic cores were described by projector-augmented wave (PAW) pseudopotentials^62^ with an energy cutoff of 500 eV. A first-order, Methfessel-Paxton smearing of 0.05 eV was applied to the orbital occupation during geometry optimization and for energy computations.

Next, the adsorption energies on different structures were evaluated using four-layer 2 × 2 supercells with the bottom two layers constrained. In addition, [6 × 6 × 1] Monkhorst-Pack *k*-point grids were also used with a convergence threshold of 10^−5^ eV for the iteration in the self-consistent field (SCF)^63^. Structural optimizations were kept until force components were <0.02 eV Å^−1^. Phonon modules in VASP 5.3 code were used to compute the vibrational frequencies of free molecules and adsorbates. Free energy corrections, including correction of the effect from zero-point energy, pressure, inner energy, and entropy, were determined by applying a standard thermodynamic correction.

## Supplementary information

Supplementary Information

Peer review file

## Data Availability

Data that support findings from this study are available from the corresponding author on reasonable request.
