# Supplementary material for: Dynamic rhenium dopant boosts ruthenium oxide for durable oxygen evolution
Source: Nat Commun. 2023 Jan 21;14:354. doi: 10.1038/s41467-023-35913-6 (PMC9867741; doi:10.1038/s41467-023-35913-6)
Supplement: Supplementary file 1 — Supplementary Information [file 41467_2023_35913_MOESM1_ESM.pdf]

Supplementary Information for

Dynamic rhenium dopant boosts ruthenium oxide for durable oxygen evolution

Huanyu Jin[1, 2, 8], Xinyan Liu[3, 8], Pengfei An[4], Cheng Tang[1], Huimin Yu[5], Qinghua Zhang[6], Hong-Jie Peng[3], Lin Gu[6], Yao Zheng[1], Taeseup Song[7], Kenneth Davey[1], Ungyu Paik[7], Juncai Dong[4, *], and Shi-Zhang Qiao[1, *]

[1] School of Chemical Engineering and Advanced Materials, The University of Adelaide, Adelaide, SA 5005, Australia

[2] Institute for Sustainability, Energy and Resources, The University of Adelaide, Adelaide, SA 5005, Australia

[3] Institute of Fundamental and Frontier Sciences, University of Electronic Science and Technology of China, Chengdu 611731, Sichuan, China

[4] Beijing Synchrotron Radiation Facility, Institute of High Energy Physics, Chinese Academy of Sciences, Beijing 100049, China

[5] Future Industries Institute, University of South Australia, Mawson Lakes Campus, Adelaide, SA 5095, Australia

[6] Beijing National Laboratory for Condensed Matter Physics, Institute of Physics, Chinese Academy of Sciences, Beijing 100190, China

[7] Department of Energy Engineering, Hanyang University, Seoul 04763, Republic of Korea

[8] These authors contributed equally: Huanyu Jin, Xinyan Liu.

E-mail: dongjc@ihep.ac.cn; s.qiao@adelaide.edu.au

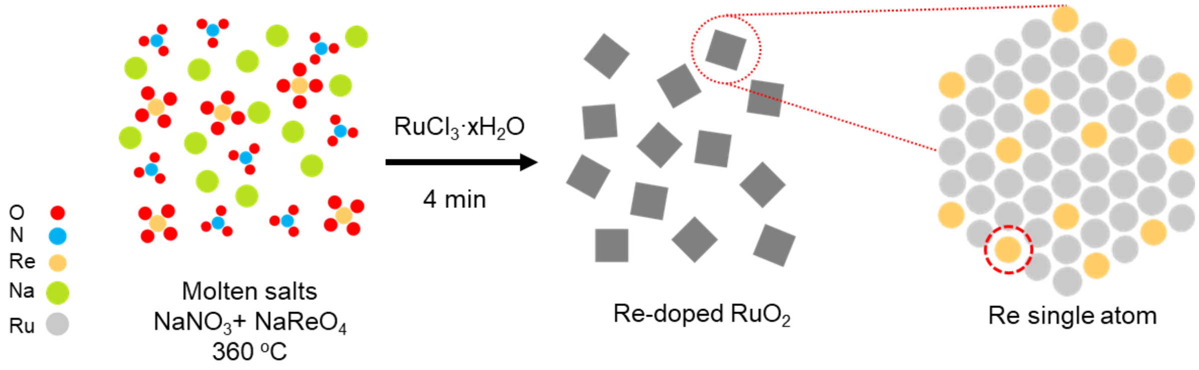

Supplementary Fig. 1 | Materials synthesis. Schematic for molten-salt synthesis of Re-RuO_2.

The $NaNO_3$ and $NaReO_4$ mixture are heated in a muffle-furnace to 360 °C until the mix melts. $RuCl_3 \cdot xH_2O$ is added to react for 5 min. Growth of Re-RuO_2 involves Ru^{3+} as a reductant and ReO_4^- and NO_3^- as oxidizers. $NaNO_3$ provides an oxidative environment to ensure complete oxidation of Ru^{3+} to Re-RuO_2 nanoparticles. Loading mass of Re is controlled via concentration of sodium perrhenate.

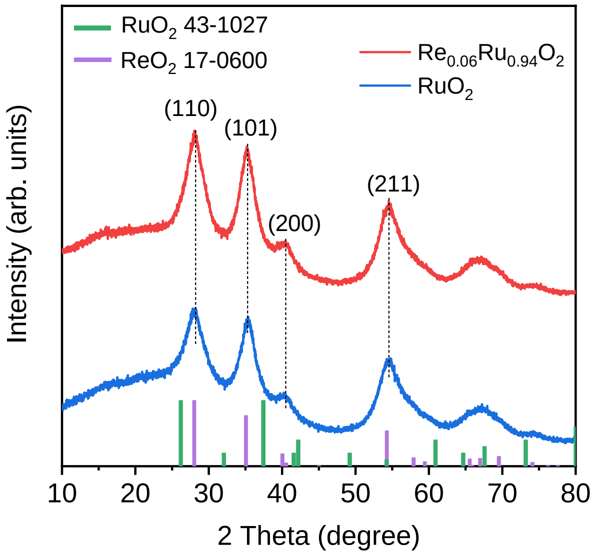

Supplementary Fig. 2 | Structure characterization. XRD pattern for $Re_{0.06}Ru_{0.94}O_2$ and RuO_2. Both samples exhibit a rutile phase. No ReO_x peak is detected.

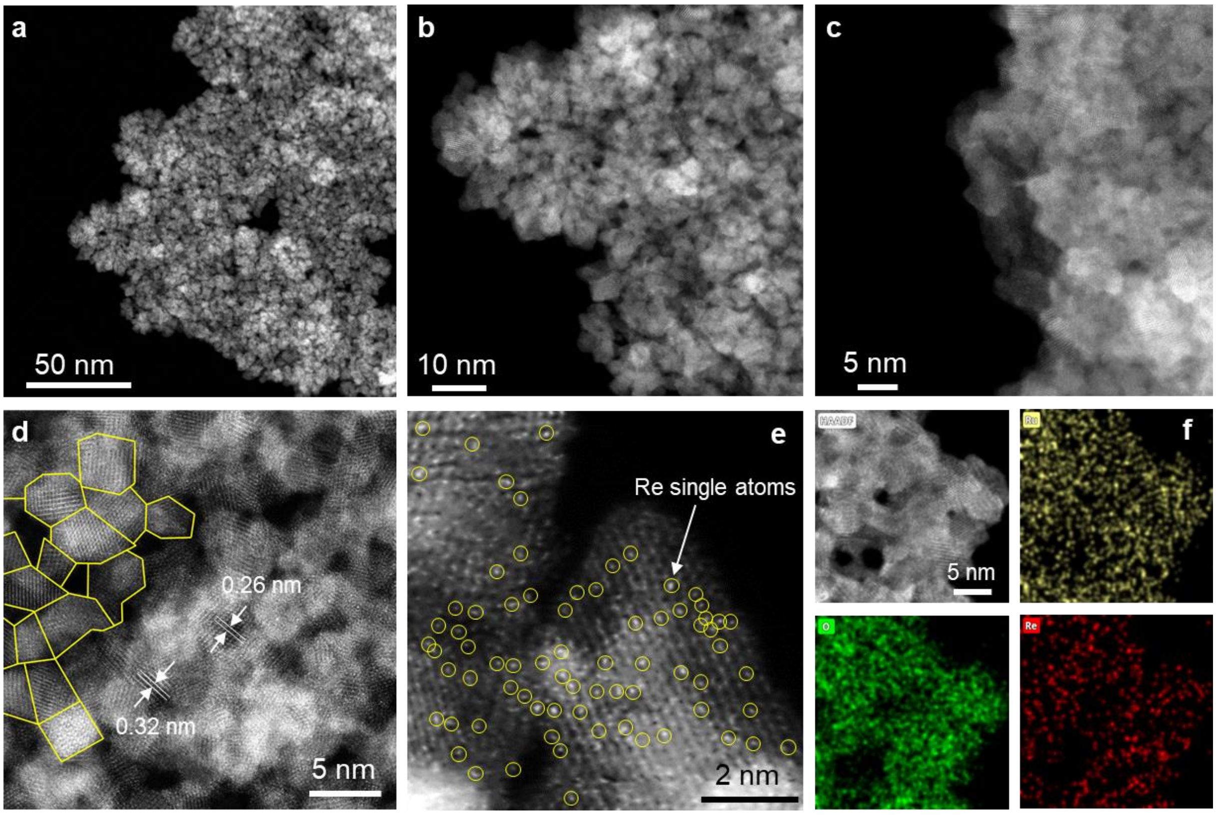

Supplementary Fig. 3 | Atomic structure characterization of pristine $Re_{0.06}Ru_{0.94}O_2$. Low-resolution STEM images of **a-d** pristine $Re_{0.06}Ru_{0.94}O_2$. Nanocrystal catalyst with grain size 2 to 5 nm. Crystal structure of $Re_{0.06}Ru_{0.94}O_2$ is a rutile phase in accordance with XRD data. **e**, High-resolution STEM image of pristine $Re_{0.06}Ru_{0.94}O_2$ confirms Re single atoms are dispersed uniformly in RuO_2 lattice. **f**, EDS mapping of pristine $Re_{0.06}Ru_{0.94}O_2$.

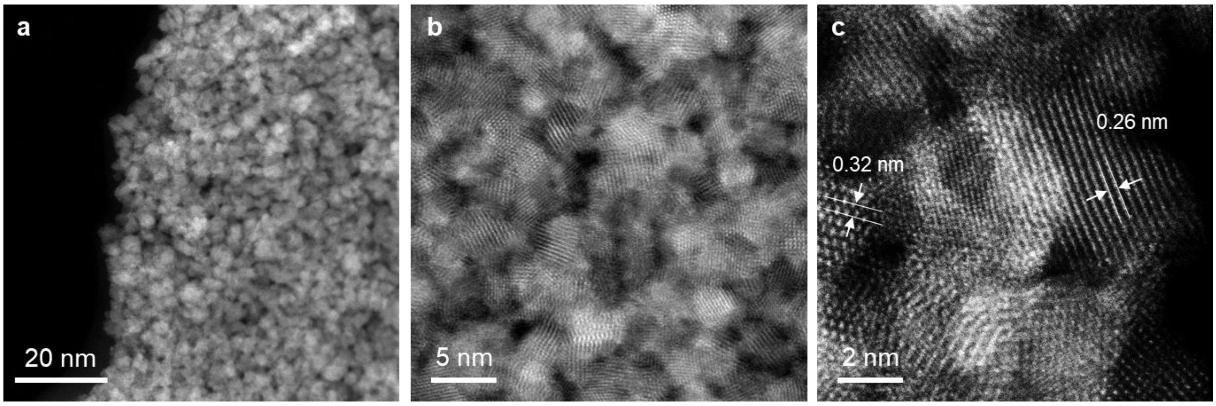

Supplementary Fig. 4 | Atomic structure characterization of pristine RuO_2. Low and high-resolution STEM images of pristine RuO_2.

HAADF-STEM images confirm $Re_{0.06}Ru_{0.94}O_2$ has similar atomic arrangement with RuO_2 (**Supplementary Figs. 3 and 4**), evidencing Re doping does not change rutile crystal structure. Re dopants are distributed in the RuO_2 lattice uniformly without bulk phase Re oxides formation (**Supplementary Fig. 3**).

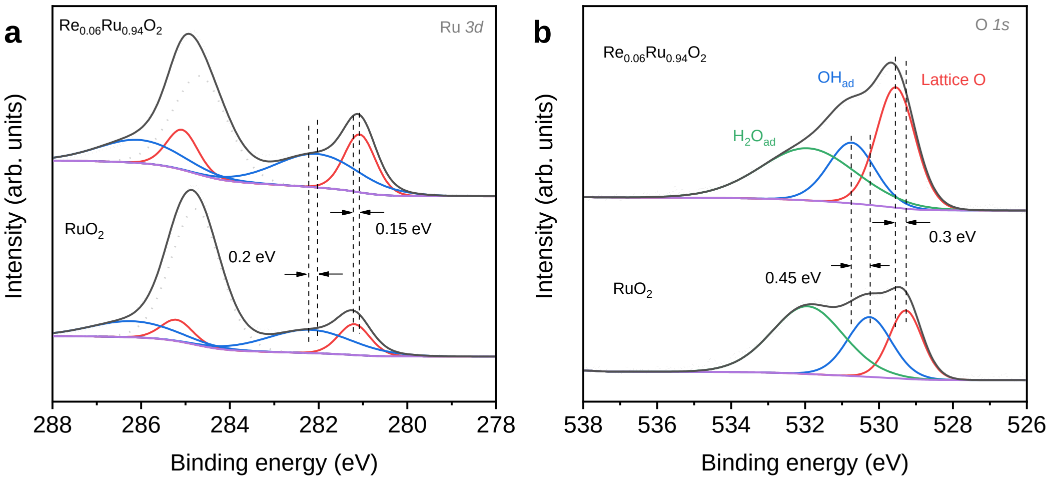

Supplementary Fig. 5 | XPS characterization of pristine RuO_2 and $Re_{0.06}Ru_{0.94}O_2$. a, Ru *3d* and **b**, O *1s* XPS spectra pristine RuO_2 and $Re_{0.06}Ru_{0.94}O_2$. Grey-colour peak at 284.6 eV in **a** is C *1s* signal.

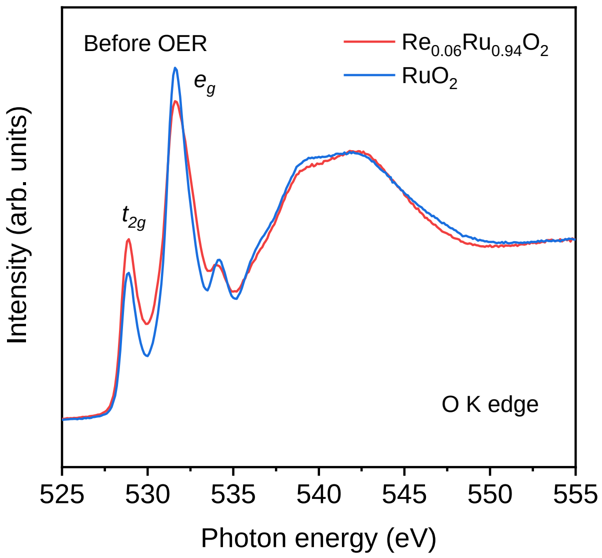

Supplementary Fig. 6 | Synchrotron NEXAFS characterizations. O K-edge NEXAFS spectra for pristine RuO_2 and $Re_{0.06}Ru_{0.94}O_2$. The sharp peak at *ca*. 528 eV refers to t_{2g} states and is followed by a broader peak at *ca*. 533 eV related to e_g states. The two 'sharp' features denoted are attributable to the excitation of the O *1s* core electrons into hybridized states between O *2p* and Ru 4*d* t_{2g} and e_g states because of splitting by the crystal field.

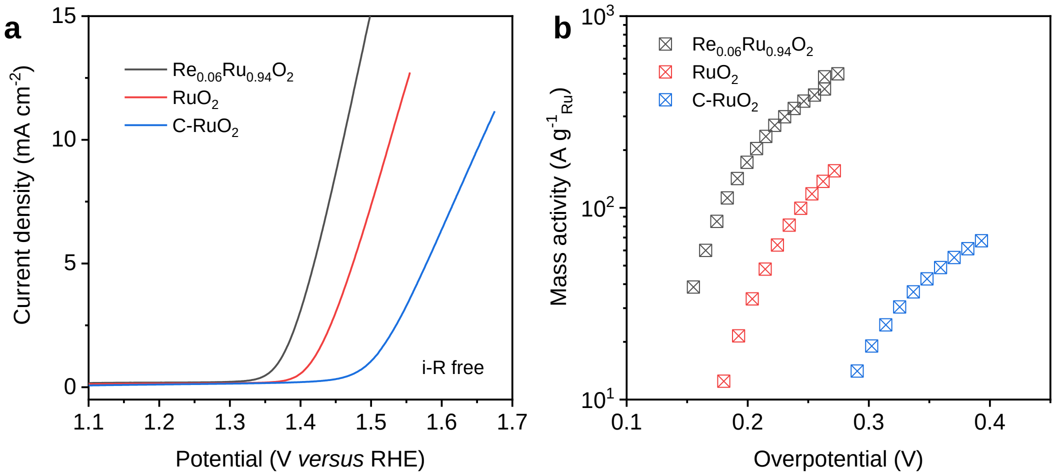

Supplementary Fig. 7 | Intrinsic activity of catalysts. a, LSV curves of different catalysts without i-R compensation. **b**, Mass activity of $Re_{0.06}Ru_{0.94}O_2$, RuO_2 and C-RuO_2.

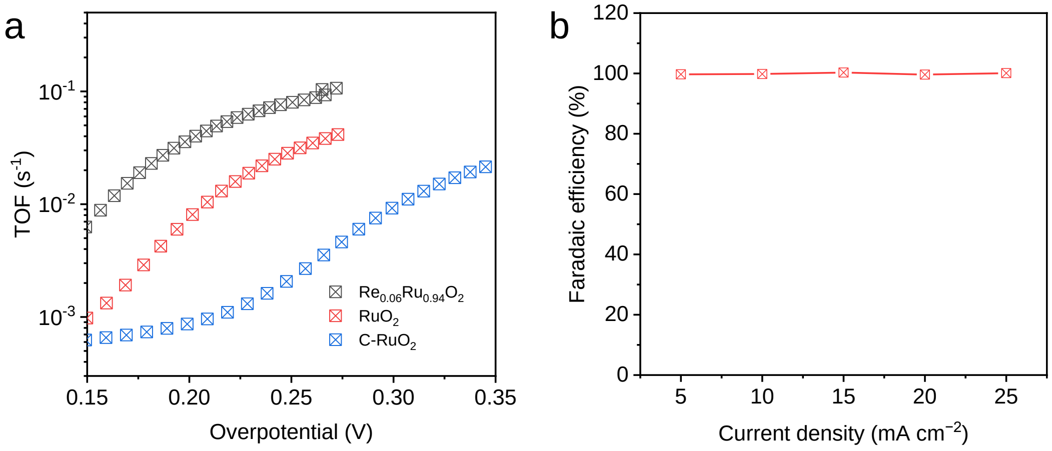

Supplementary Fig. 8 | a, TOF for $Re_{0.06}Ru_{0.94}O_2$, RuO_2 and C-RuO_2. **b**, OER Faradaic efficiency at different current densities for $Re_{0.06}Ru_{0.94}O_2$.

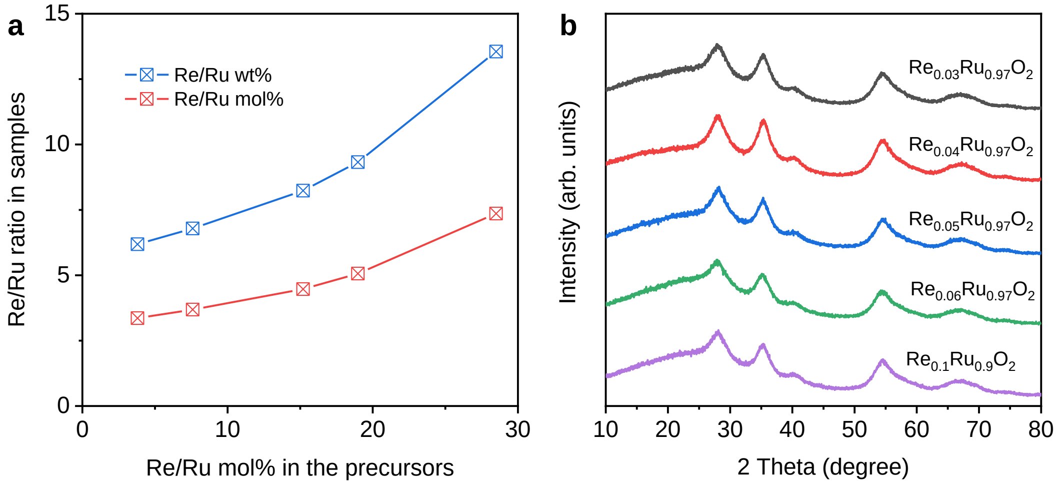

Supplementary Fig. 9 | Stoichiometric characterization. a, Re and Ru ratio in samples with the change of Re and Ru ratio in precursors. **b**, XRD pattern for Re-RuO_2 samples with differing Re.

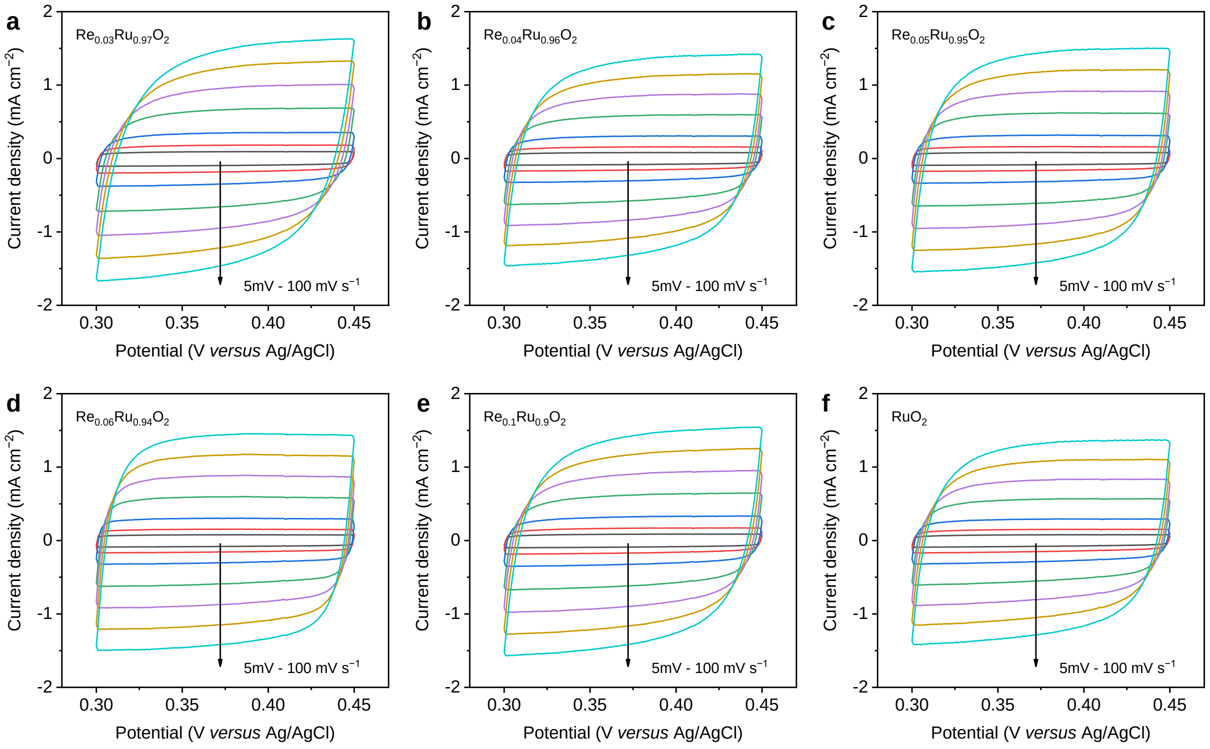

Supplementary Fig. 10 | ECSA for different catalyst. CV curves for samples for ECSA of Re-RuO_2 with differing Re and RuO_2.

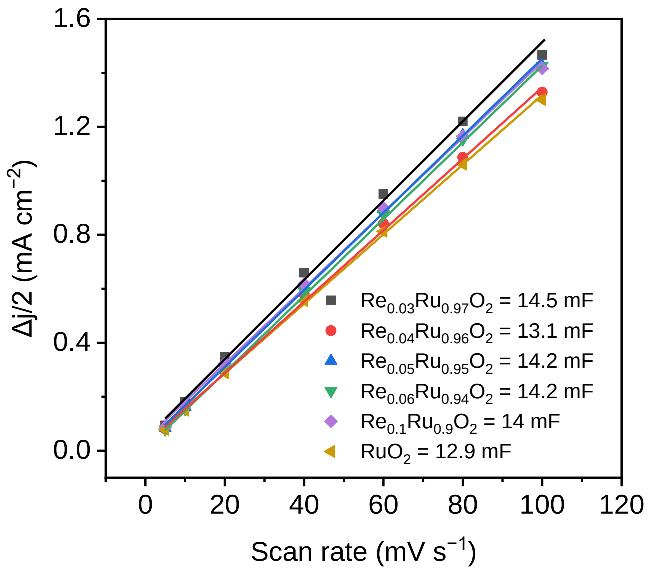

Supplementary Fig. 11 | ECSA for catalysts. Double-layer capacitance (C_{dl}) for catalysts as represented by curve-slope.

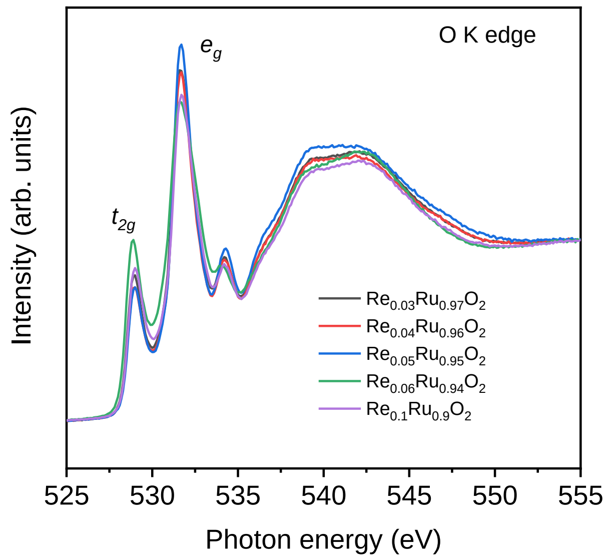

Supplementary Fig. 12 | Synchrotron NEXAFS characterization of Re-RuO_2. O K-edge NEXAFS spectra for Re-RuO_2 with differing Re.

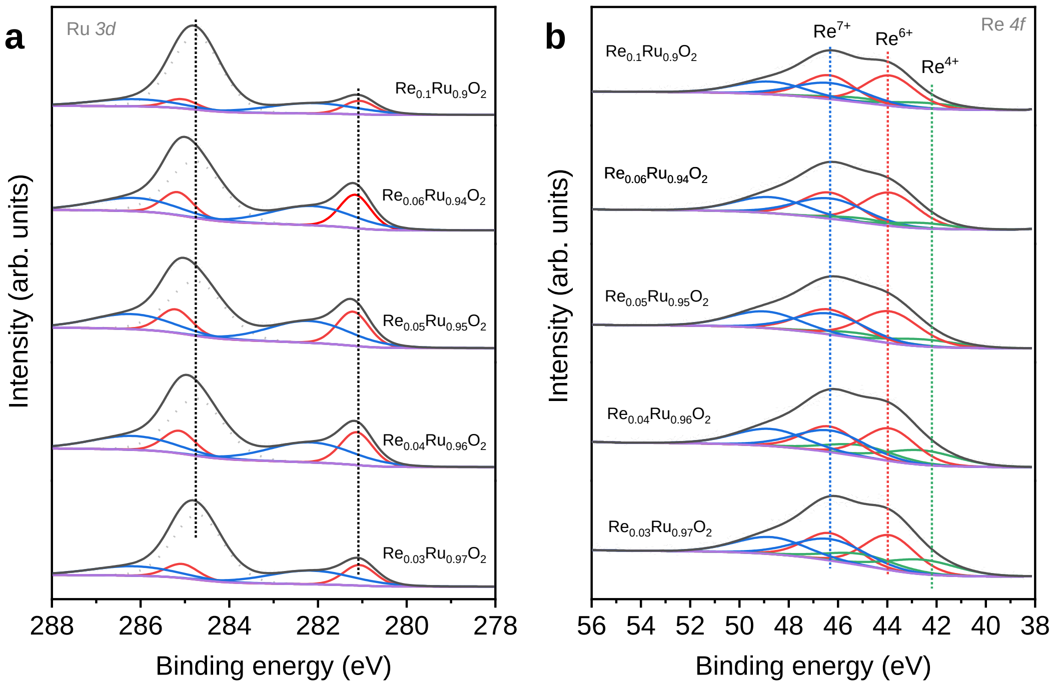

Supplementary Fig. 13 | XPS characterization of Re-RuO_2. **a**, Ru *3d* and **b**, Re *4f* XPS spectra of Re-RuO_2 with differing Re. Grey-colour peak at 284.6 eV in **a** is C *1s* signal.

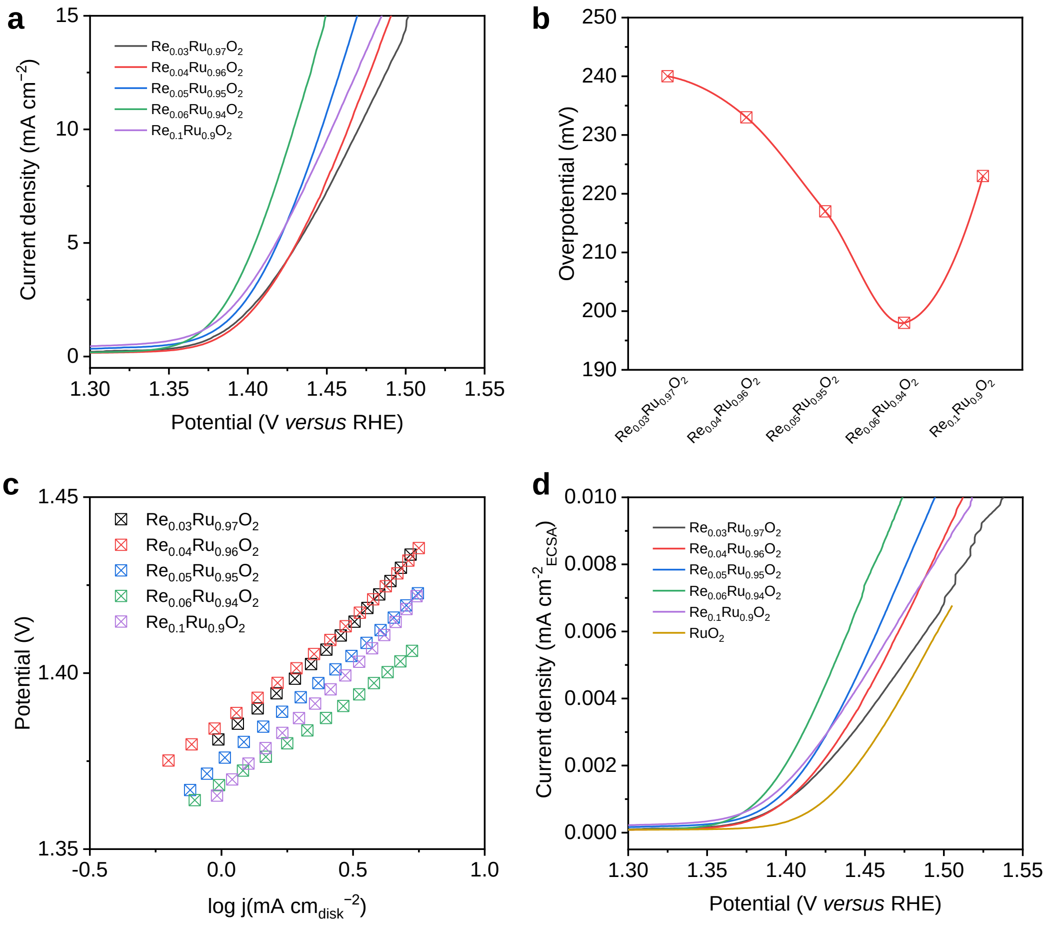

Supplementary Fig. 14 | OER for Re-RuO_2. a, LSV curves for Re-RuO_2 with differing Re in O_2-saturated 0.1 M $HClO_4$. **b**, η_{10} for Re-RuO_2 electrocatalyst. **c**, Tafel plot for Re-RuO_2 with differing Re corresponding with **a**. **d**, LSV curves of different catalysts normalized to ECSA.

Additionally, the impact of Re doping on OER for Re-RuO_2 was determined. The Re doping level is controlled by the amount of $NaReO_4$ in the molten salt (**Supplementary Fig. 9**). Re doping does not change crystal structure and electrochemical active surface area (ECSA), however, it changes the electronic structure of RuO_2 (**Supplementary Figs. 9-13**). As is presented in **Supplementary Fig. 14**, the relationship between activity and Re doping level in Re-RuO_2 is a volcano plot. $Re_{0.06}Ru_{0.94}O_2$ exhibited the 'best' performance with the lowest η_{10} and Tafel slope. This is because Re dopants are inert for OER, compared to Ru site. High doping levels lower the density of the active site on the surface and, decrease overall performance.

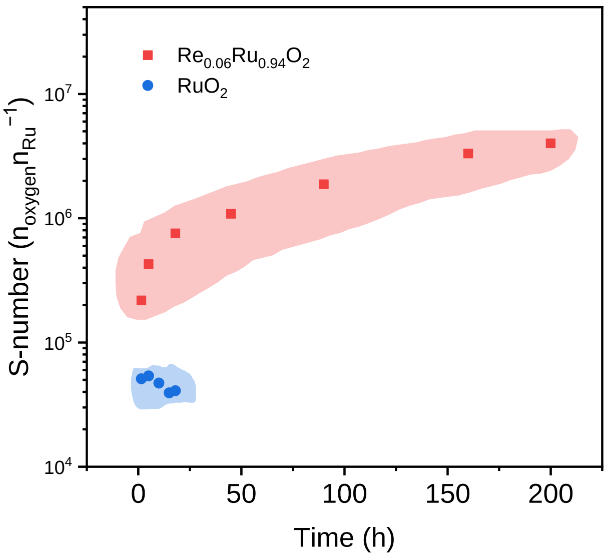

Supplementary Fig. 15 | Durability of catalysts. S-number for $Re_{0.06}Ru_{0.94}O_2$ and RuO_2 with change of reaction time.

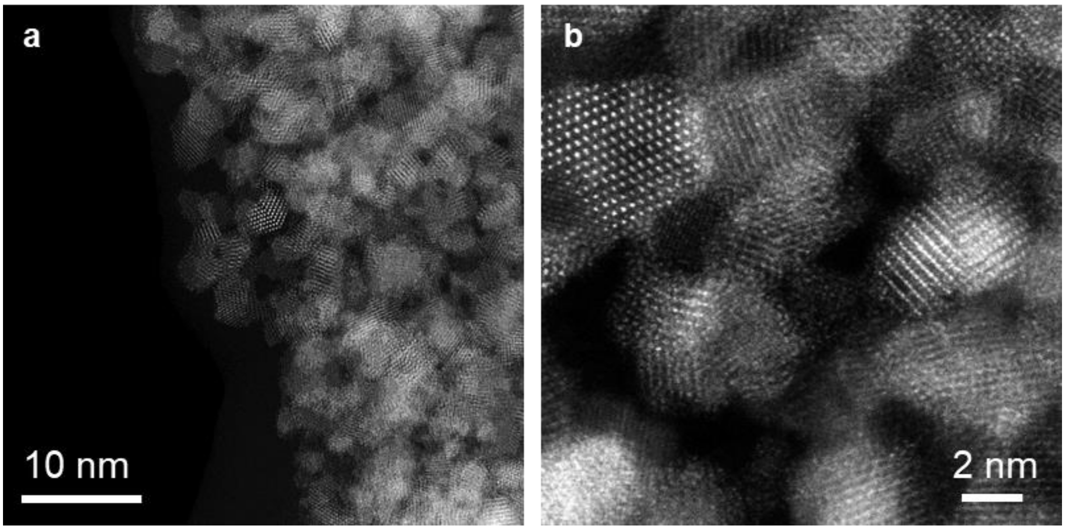

Supplementary Fig. 16 | Atomic structure of pristine RuO_2 after OER. **a**, Low and **b**, high-resolution HAADF-STEM images of RuO_2 after stability test for 20 h.

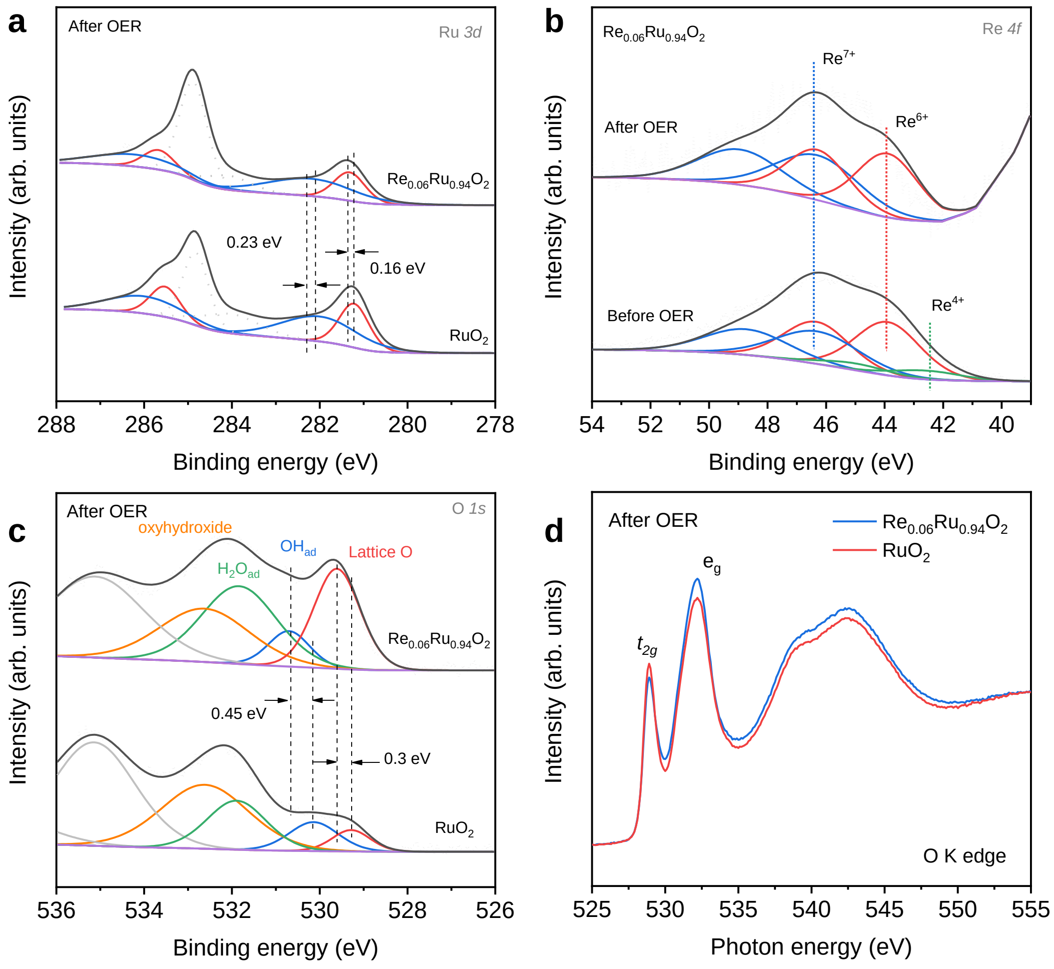

Supplementary Fig. 17 | Post-reaction XPS and NEXAFS characterizations. a, Ru *3d* XPS spectra for post-reaction RuO_2 (20 h) and $Re_{0.06}Ru_{0.94}O_2$ (50 h). Grey-colour peak at 284.6 eV and in **a** is C *1s* signal. **b**, Re *4f* XPS spectra for $Re_{0.06}Ru_{0.94}O_2$ before and after 50 h OER test. **c**, O *1s* XPS spectra and **d**, O K-edge NEXAFS spectra for post-reaction RuO_2 (20 h) and $Re_{0.06}Ru_{0.94}O_2$ (50 h). Grey peak at 535 eV is for O in Nafion in **c**.

XPS spectra for samples after OER were analyzed because the surface oxygen vacancies on RuO_2-based electrocatalysts usually generate more adsorbed oxygen species (OH_{ad}) than catalysts with AEM pathway after stability testing[37]. As is seen in **Supplementary Fig 17c**, the O *1s* spectra for RuO_2 and $Re_{0.06}Ru_{0.94}O_2$ after stability testing exhibit four (4) prominent peaks *ca.* 529.6 , 530.6 , 531.9 and 532.7 eV, which are attributed to, respectively, lattice oxygen, OH_{ad}, adsorbed H_2O and oxyhydroxide. Both samples contain Ru oxyhydroxide that generated during OER through AEM pathway. However, the RuO_2 after OER exhibits a greater percentage of OH_{ad} compared with pristine matrix and $Re_{0.06}Ru_{0.94}O_2$, evidencing the greater O-vacancy density on RuO_2 via a LOM-AEM pathway.

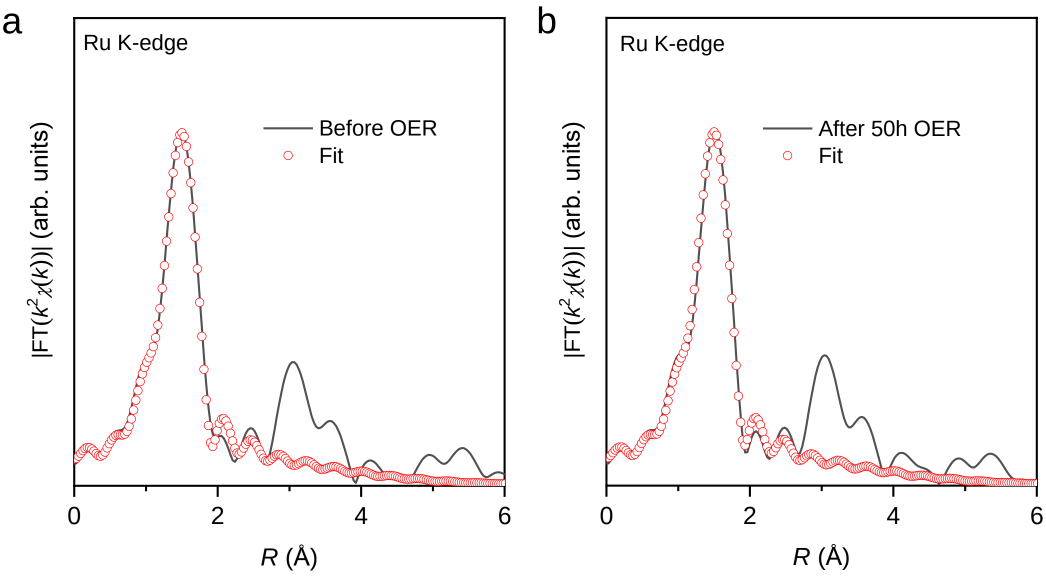

Supplementary Fig. 18 | Fitted data for Ru K-edge of $Re_{0.06}Ru_{0.94}O_2$ before and after OER. Ru K-edge EXAFS fitting analyzes for $Re_{0.06}Ru_{0.94}O_2$ before and after 50 h stability test. Best-fit parameters are given in **Supplementary Table 3**.

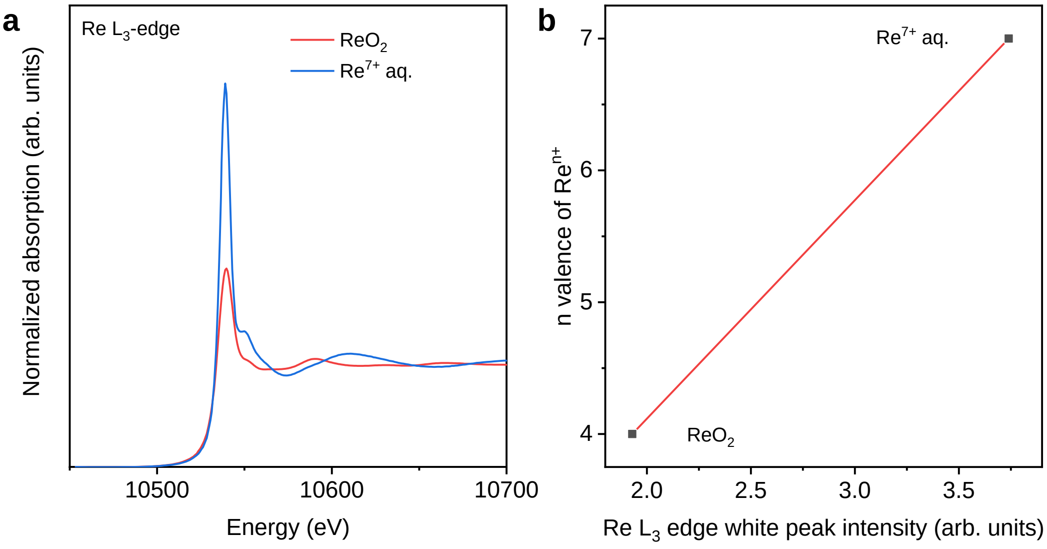

Supplementary Fig. 19 | XAS chemical valence calibration. a, Re L_3-edge of ReO_2 and Re^{7+} species in aqueous solution. **b**, Calibration of Re chemical valence against the relative edge white peak intensity deduced from the reference materials in **a**.

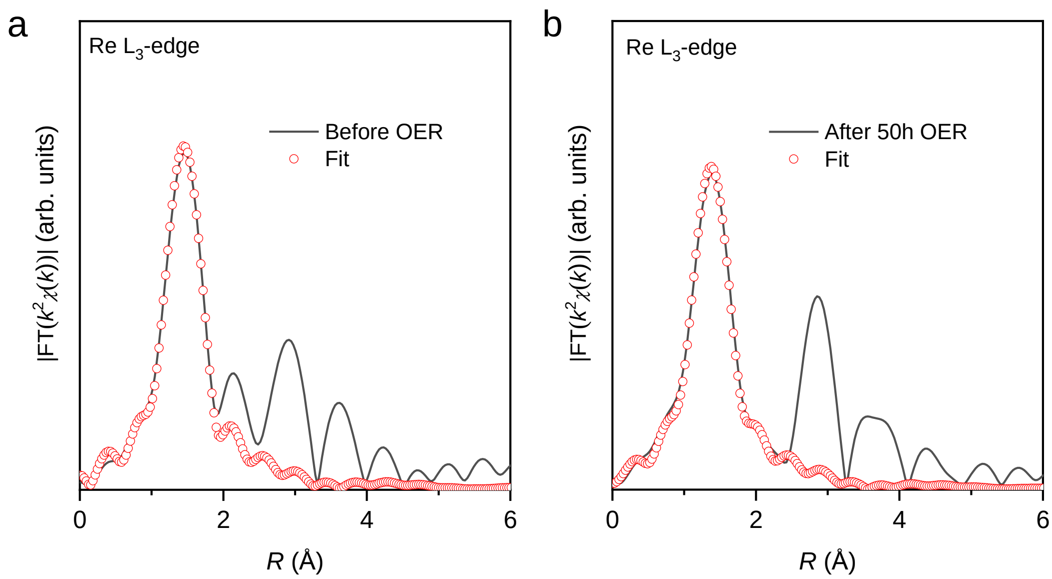

Supplementary Fig. 20 | Fitted data for Re L_3-edge of $Re_{0.06}Ru_{0.94}O_2$ before and after OER. Re L_3-edge EXAFS fitting analyzes for $Re_{0.06}Ru_{0.94}O_2$ before and after 50 h stability test. Best-fit parameters are given in **Supplementary Table 4**.

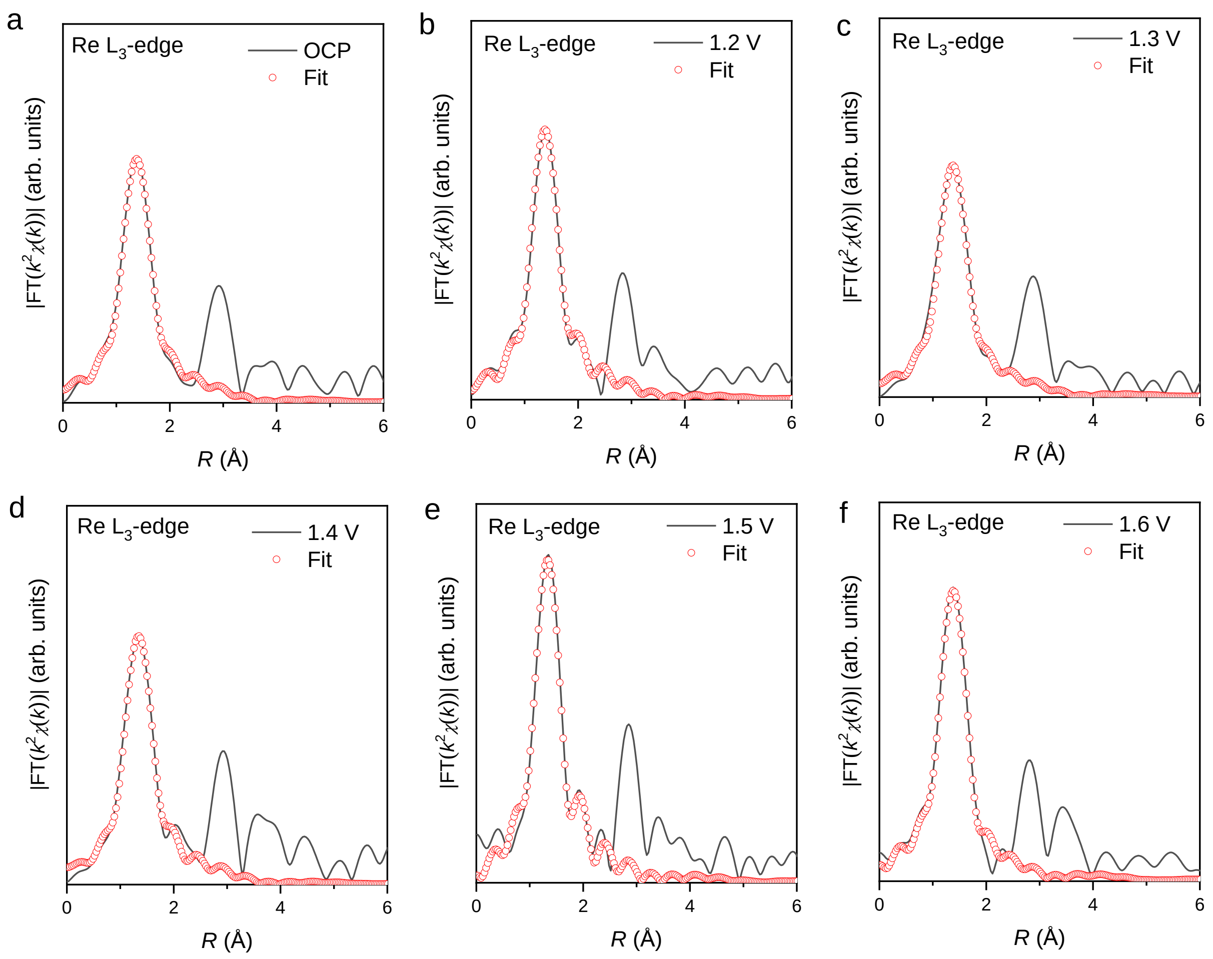

Supplementary Fig. 21 | Fitted data for operando XAS. Operando Re L_3-edge EXAFS fitting analyzes for $Re_{0.06}Ru_{0.94}O_2$ at different applied potentials. Measured and computed spectra are in good agreement. Best-fit parameters are given in **Supplementary Table 4**.

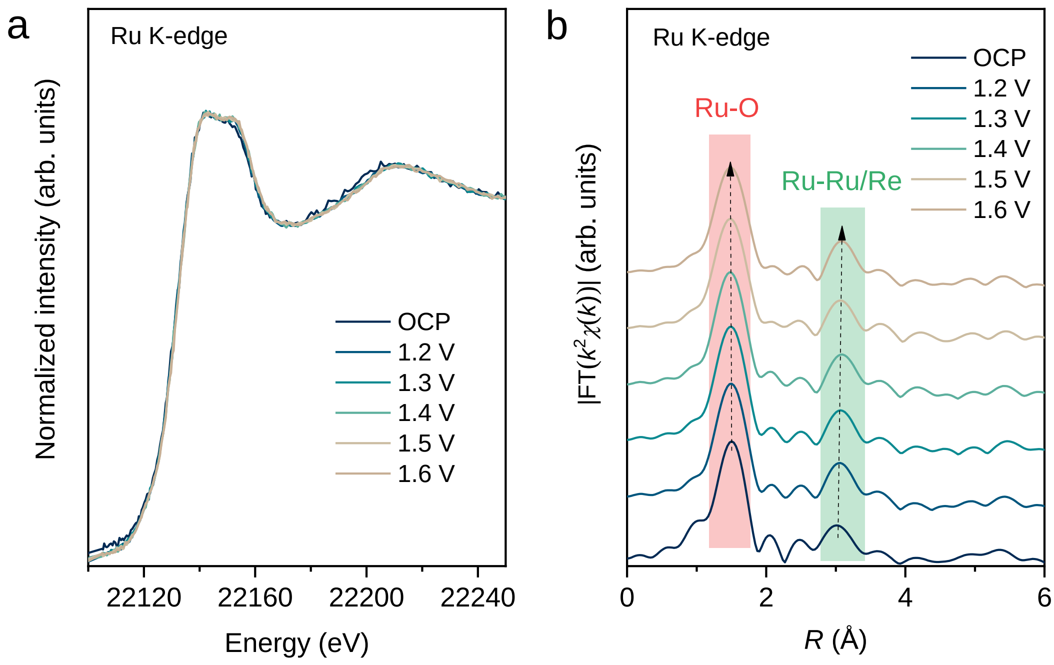

Supplementary Fig. 22 | Operando XAS characterization of Ru K-edge for $Re_{0.06}Ru_{0.94}O_2$. **a**, Ru K-edge XANES spectra for $Re_{0.06}Ru_{0.94}O_2$ at differing potentials in O_2-saturated 0.1 M $HClO_4$. **b**, FT-EXAFS signals for $Re_{0.06}Ru_{0.94}O_2$ corresponding to **a**.

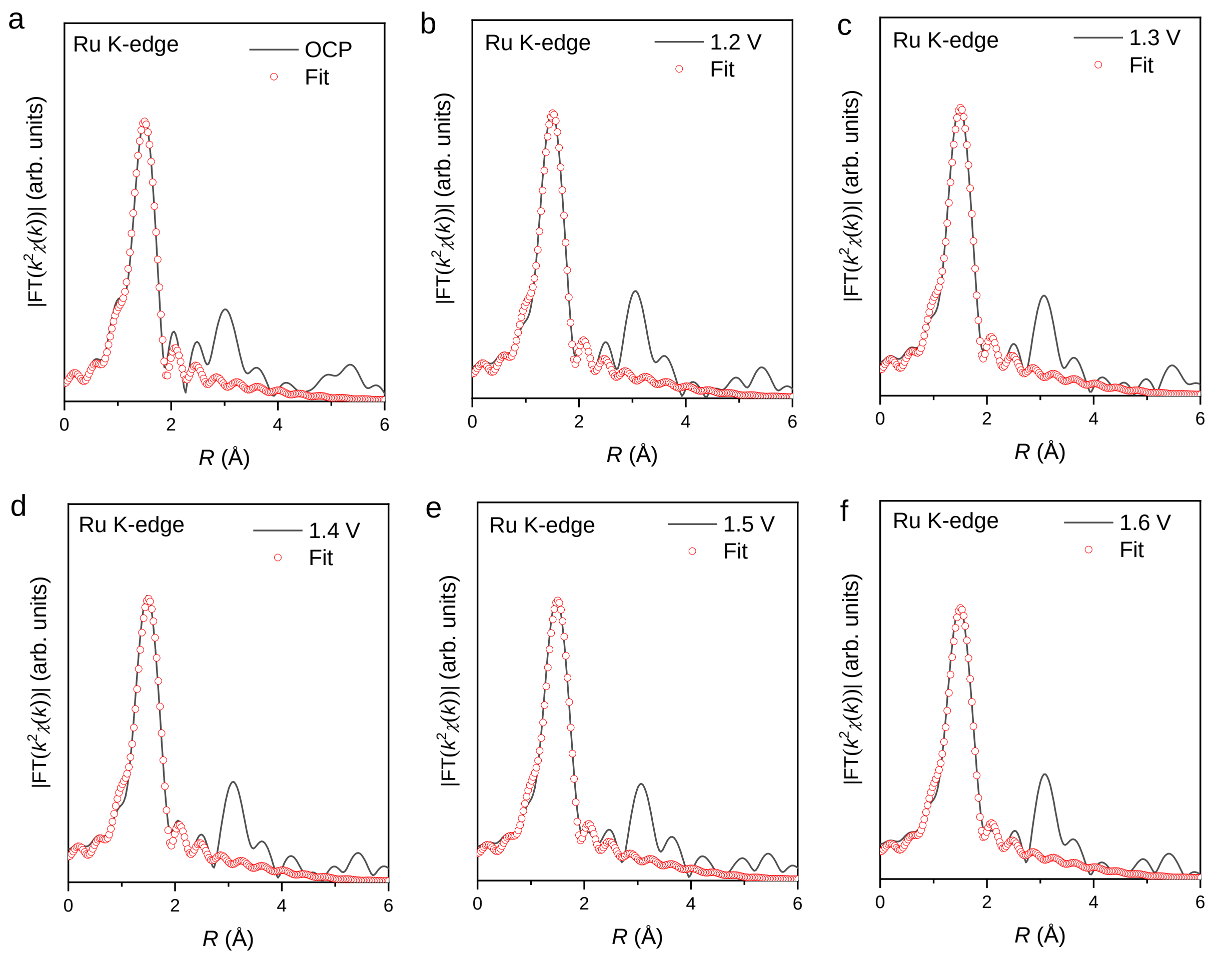

Supplementary Fig. 23 | Fitted data for operando XAS. Operando Ru K-edge EXAFS fitting analyzes for $Re_{0.06}Ru_{0.94}O_2$ at different applied potentials. Measured and computed spectra are in good agreement. Best-fit parameters are given in **Supplementary Table 3**.

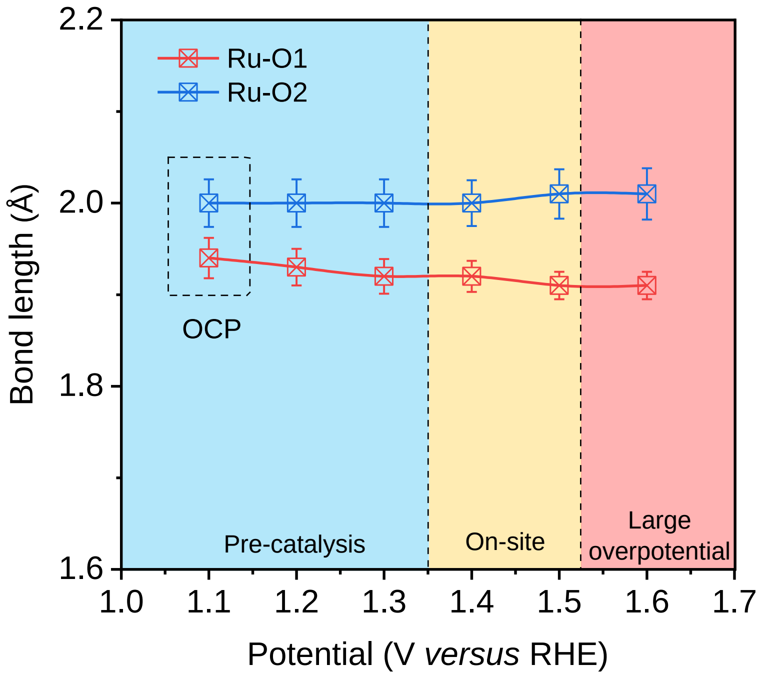

Supplementary Fig. 24 | Change in bond length for Ru–O1 and Ru–O2 coordination shells.

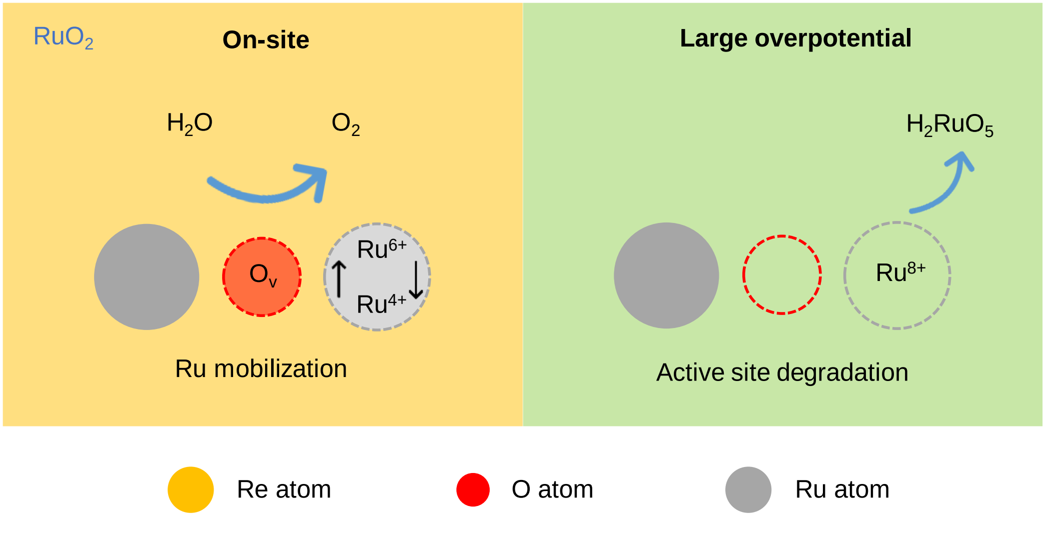

Supplementary Fig. 25 | Schematic for comparison of RuO_2 catalyst durability in acidic OER without Re dopants. The Rutile RuO_2 decays rapidly during OER in acid because of the loss of catalytic active sites.

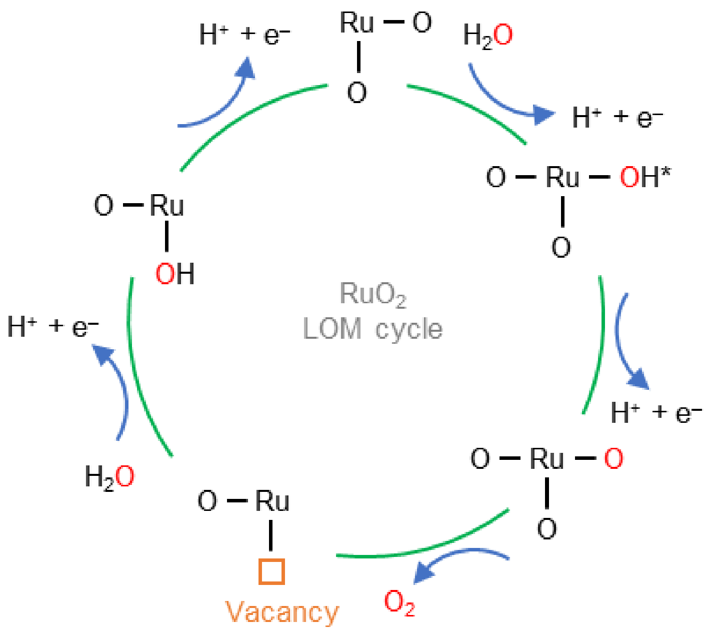

Supplementary Fig. 26 | Illustration for LOM pathway on RuO_2 toward acidic OER.

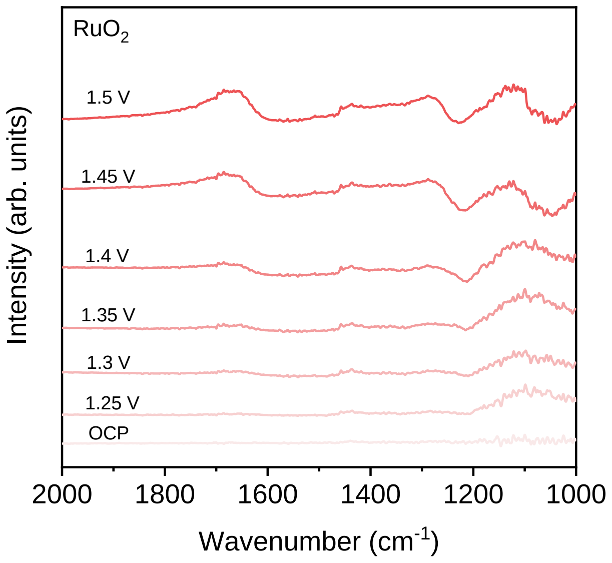

Supplementary Fig. 27 | In situ ATR-SEIRAS spectra for RuO_2 recorded during multi-potential steps.

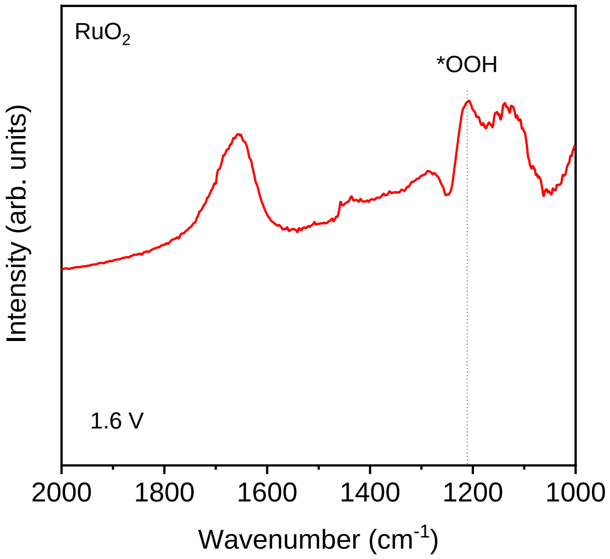

Supplementary Fig. 28 | Reaction pathway on RuO_2 at large overpotential. In situ ATR-SEIRAS spectrum for RuO_2 at the potential of 1.6 V. Typical *OOH intermediate in AEM pathway appears on RuO_2 at large overpotential.

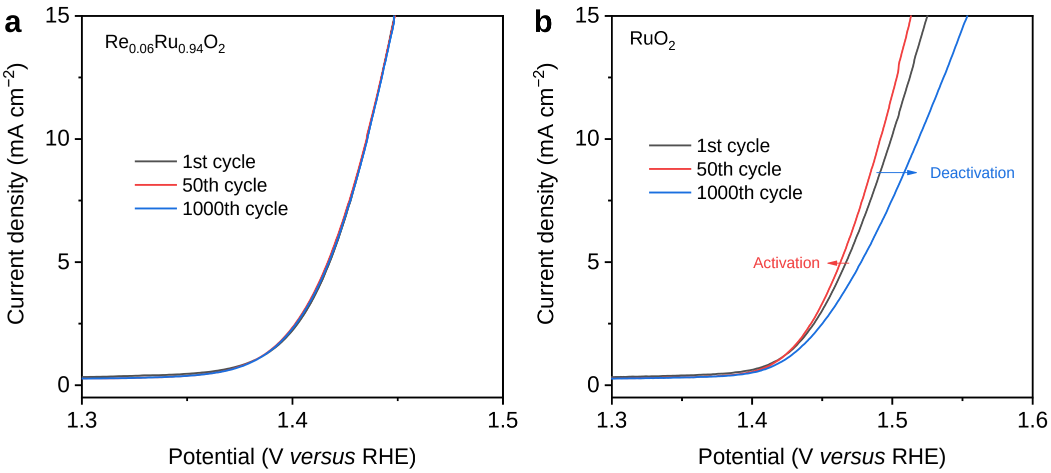

Supplementary Fig. 29 | Stability test to confirm reaction pathway for of catalysts. LSV curves for **a** $Re_{0.06}Ru_{0.94}O_2$ and **b** RuO_2 following 1, 50 and 1000 CV scans.

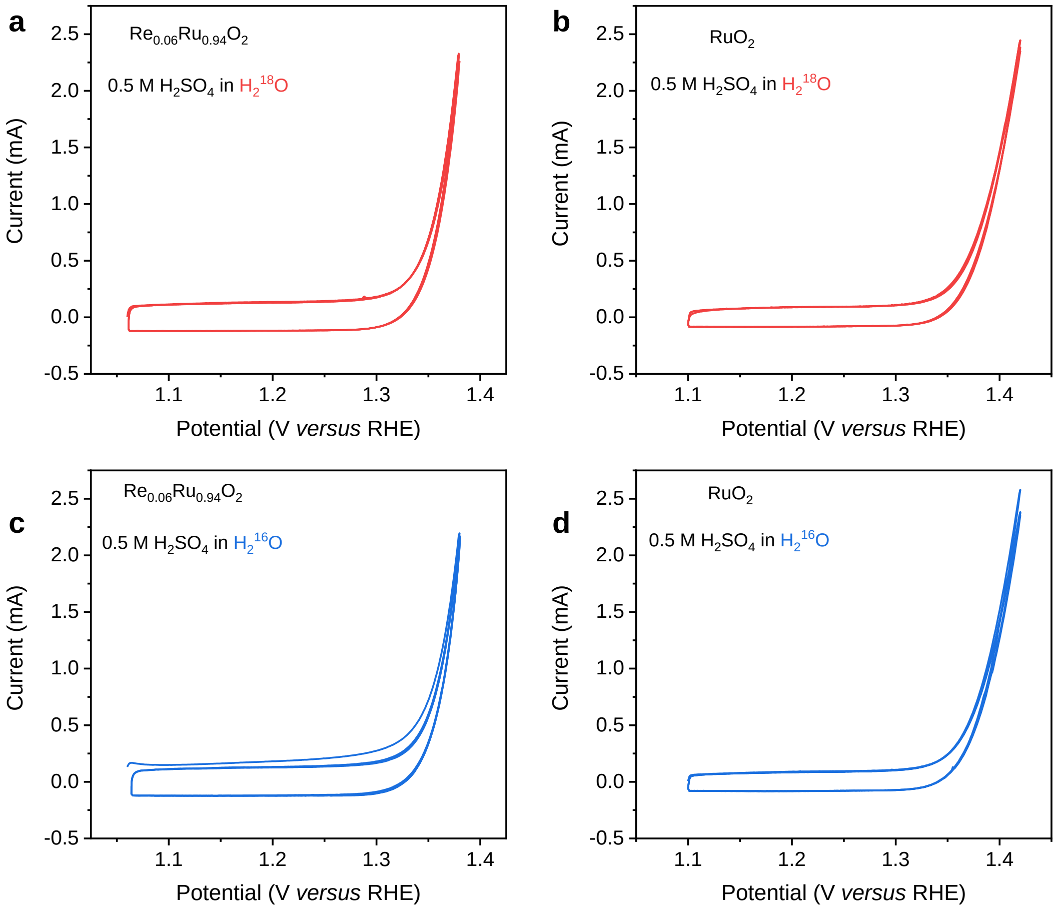

Supplementary Fig. 30 | Electrochemical performance for RuO_2 and $Re_{0.06}Ru_{0.94}O_2$ during DEMS test. CV curves for $Re_{0.06}Ru_{0.94}O_2$ and RuO_2 in DEMS electrochemical cell with 0.05 M H_2SO_4 in $H_2{}^{18}O$ and $H_2{}^{16}O$ water feedback.

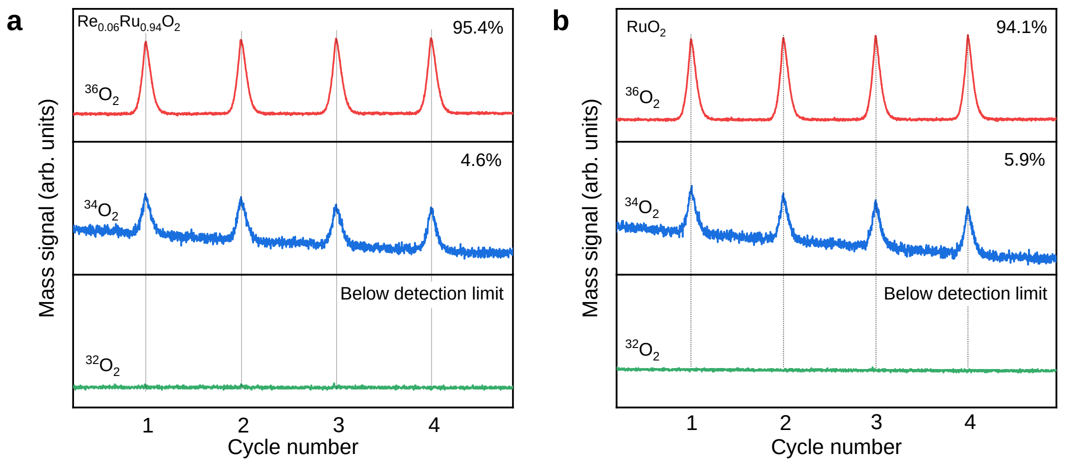

Supplementary Fig. 31 | DEMS test in $H_2{}^{18}O$ aqueous sulphuric acid electrolyte. DEMS signals for $^{36}O_2$, $^{34}O_2$ and $^{32}O_2$ from the reaction products for $Re_{0.06}Ru_{0.94}O_2$ and RuO_2 catalysts in $H_2{}^{18}O$ aqueous sulphuric acid electrolyte.

Two-step DEMS experiments using $H_2{}^{18}O$ and $H_2{}^{16}O$ in 0.05 M H_2SO_4 electrolyte were used. In DEMS measurement, 98 % H_2SO_4 is used (instead of 70 % $HClO_4$) as electrolyte to minimize the influence of $H_2{}^{16}O$. 1) $Re_{0.06}Ru_{0.94}O_2$ and RuO_2 were loaded onto a Au-coated, porous polytetrafluoroethylene (PTFE) membrane working electrode and subjected to four cyclic voltammetry (CV) cycles in 0.05 M H_2SO_4-$H_2{}^{18}O$ electrolyte (**Supplementary Fig. 26**). **Supplementary Fig 27** presents recorded mass signal for OER gaseous products in 0.05 M H_2SO_4-$H_2{}^{18}O$. At each CV scan, $Re_{0.06}Ru_{0.94}O_2$ (steadily) yielded 4.6 % $^{34}O_2$ and 95.4 % $^{36}O_2$. The $^{34}O_2$ originates from the surface oxygen adsorbates and impurity in the electrolyte (97 % $H_2{}^{18}O$). In contrast, RuO_2 yielded 5.9 % $^{34}O_2$ and 94.1 % $^{36}O_2$, which is 1.3 % greater than the $^{34}O_2$ yielded by $Re_{0.06}Ru_{0.94}O_2$. Significantly, no apparent (distinguishable) $^{32}O_2$ signal was observed in both samples, eliminating the possibility of the OPM pathway. 2) The catalysts and the DEMS cell were washed with abundant $H_2{}^{16}O$ and dried under vacuum to remove $H_2{}^{18}O$. 3) The cell was operated in 0.05 M H_2SO_4-$H_2{}^{16}O$ electrolyte.

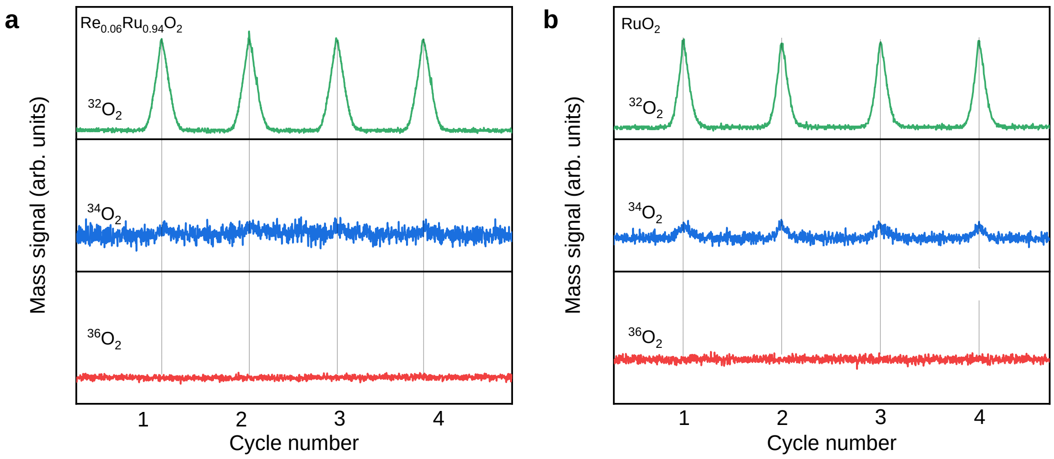

Supplementary Fig. 32 | DEMS test in $H_2{}^{16}O$ aqueous sulphuric acid electrolyte. DEMS signals for O_2 products for ^{18}O-labeled $Re_{0.06}Ru_{0.94}O_2$ and RuO_2 in electrolyte using $H_2{}^{16}O$ as solvent during four times LSV.

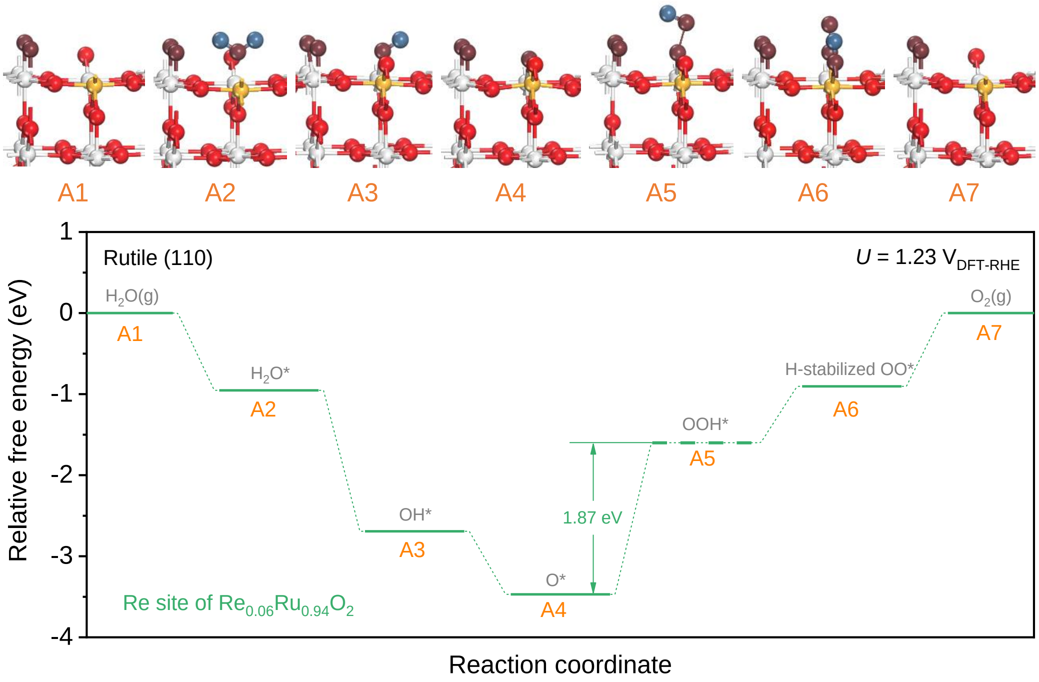

Supplementary Fig. 33 | AEM pathway on Re site for $Re_{0.06}Ru_{0.94}O_2$. Free energy diagram for OER on unsaturated Re site of $Re_{0.06}Ru_{0.94}O_2$ at 1.23 V *versus* RHE, showing the six possible intermediates for (110) surfaces. Dashed lines indicate unstable –OOH precursor states, shown as H-stabilized OO*. The significant energy difference of 1.87 eV between A4 and A5 evidences that the Re site is not active site for OER.

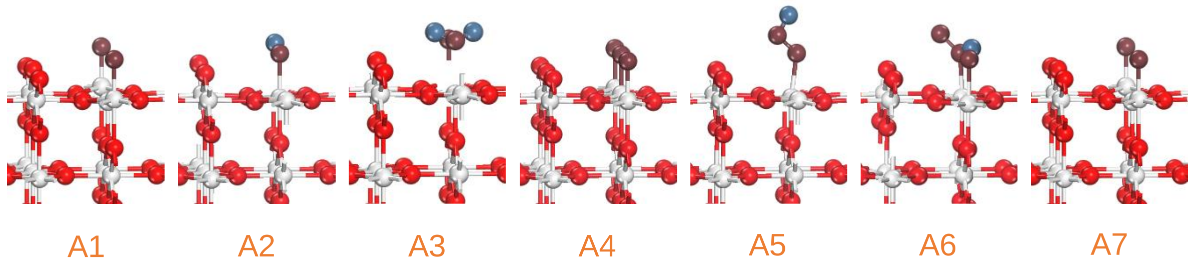

Supplementary Fig. 34 | AEM pathway on RuO_2. States A1-A7 present elementary states in AEM pathways on RuO_2.

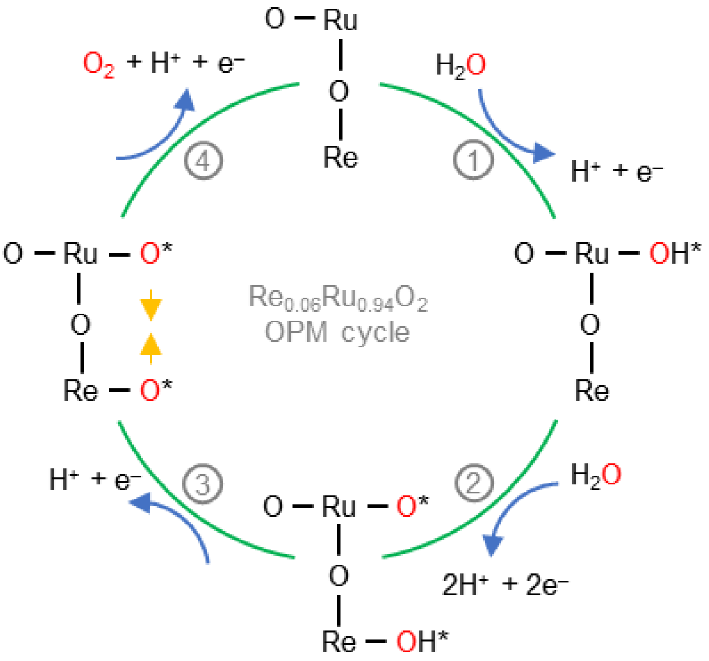

Supplementary Fig. 35 | Illustration for OPM pathway on $Re_{0.06}Ru_{0.94}O_2$ toward acidic OER.

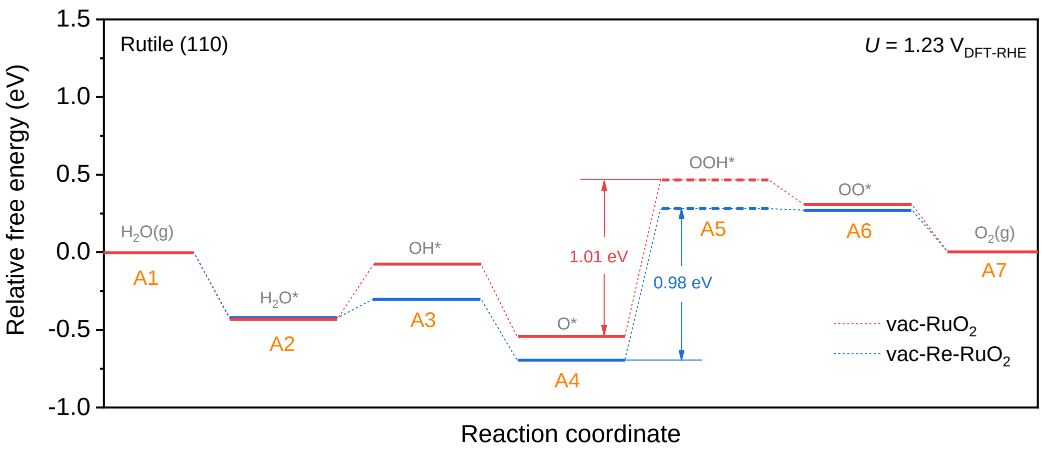

Supplementary Fig. 36 | Free energy diagram for OER on vac-RuO_2 and vac-Re-RuO_2 at 1.23 V *versus* RHE, showing the six possible intermediates for (110) surfaces. Dashed lines indicate unstable –OOH precursor states, shown as H-stabilized OO*.

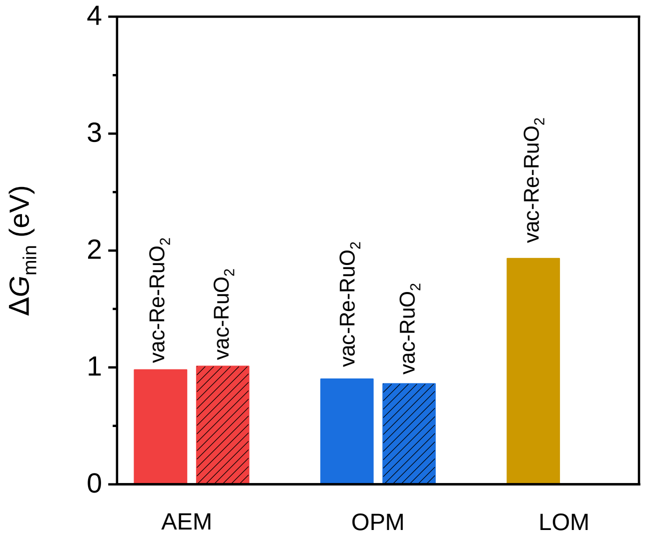

Supplementary Fig. 37 | Minimum activation energy for different reaction pathways for vac-RuO_2 and vac-Re-RuO_2.

Supplementary Table 1 | Comparison of acidic OER catalytic performance with reported noble-metal-based electrocatalysts in three-electrode system.

**Catalyst**	**Electrolyte**	**η_{10} (mV)**	**Tafel slope (mV dec^{-1})**	**Stability at η_{10} (h)**	**Mass activity $A_{noble\ metal}$ g^{-1}**	**Mass loading ($mg_{noble\ metal}$ cm^{-2})**	**Reference**
$Re_{0.06}Ru_{0.94}O_2$	0.1 M $HClO_4$	190	45.5	200	500, 7,811 (Re-Ru)[a]	0.07	This work
RuO_2	0.1 M $HClO_4$	258	50.3	18	156	0.08	This work
12Ru/MnO_2	0.1 M $HClO_4$	161	29.4	200	1,264	0.02	1
$Sr_{0.95}Na_{0.05}RuO_3$	0.1 M $HClO_4$	170	N.A.	N.A.	N.A.	0.04	2
$CaCuRu_4O_{12}$	0.5 M H_2SO_4	171	40	24	1,942	0.12	3
$Cr_{0.6}Ru_{0.4}O_2$	0.5 M H_2SO_4	178	56	10	229	0.11	4
UfD-RuO_2/CC	0.5 M H_2SO_4	179	36.9	20	N.A.	0.4	5
Cu-Doped RuO_2	0.5 M H_2SO_4	188	43.96	8	N.A.	0.19	6
SrRuIr	0.5 M H_2SO_4	190	39	1500	281	0.25	7
GB-$Ta_{0.1}Tm_{0.1}Ir_{0.8}O_{2-\delta}$	0.5 M H_2SO_4	198	64	500	3,126	0.04	8
1T-IrO_2	0.1 M $HClO_4$	197	49	126	296.8	0.73	9
$Ir_{0.7}Ru_{0.3}O_2$	0.1 M $HClO_4$	200	46	10	200	0.08	10
$(Mn_{0.8}Ir_{0.2})O_2$:10F	1.0 M H_2SO_4	200	38	24	40	0.09	11
SS Pt-RuO_2 HNSs	0.5 M H_2SO_4	228	48	100	N.A.	0.06	12
d-$ZnIr(OH)_6$ NSs/C	0.5 M H_2SO_4	252	56.1	10	1,002	0.05	13
Co-RuIr	0.1 M $HClO_4$	235	66.9	25	N.A.	0.05	14
Ru@IrO_x	0.05 M H_2SO_4	282	69.1	24	644.8	0.05	15
$Ir_{0.06}Co_{2.94}O_4$	0.1 M $HClO_4$	300	45	200	2,511	0.005	16
Ru_1-Pt_3Cu	0.1 M $HClO_4$	220	N.A.	28	779	0.016	17
Ru_1-N_4	0.5 M H_2SO_4	267	52.6	30	3,571	0.003	18

[a]Re-related Ru mass activity. Based on DFT computations, the unsaturated Ru connected to Re is the active site for OER. Therefore, the Re-related Ru mass activity is computed based on the Re-related Ru mass, and not total Ru mass in $Ru_{0.06}Ru_{0.94}O_2$.

Supplementary Table 2 | ICP-MS data for RuO_2 and $Re_{0.06}Ru_{0.94}O_2$ during stability testing.

**Time (h)**	**Ru in $Re_{0.06}Ru_{0.94}O_2$ (ppb)**	**Re in $Re_{0.06}Ru_{0.94}O_2$ (ppb)**	**Time (h)**	**RuO_2 (ppb)**
0.5	0.37	1.63	0.5	0.88
1.5	1.63	1.69	1	1.85
5	2.76	1.8	1.5	6.94
18	5.62	1.92	5	21.91
45	9.78	2.07	10	50.09
90	11.31	2.26	15	89.91
160	11.38	2.47	18	103.8
200	11.78	2.52		

Supplementary Table 3 | Ru K-edge EXAFS fitted parameters for $Re_{0.06}Ru_{0.94}O_2$[a]

**Sample**	**shell**	***N***	***R* (Å)**	**σ^2 (Å^2)**	**ΔE_0 (eV)**	**R_f, %**
$Re_{0.06}Ru_{0.94}O_2$ Pristine[b]	Ru-O1	1.8	1.92	0.003	6.5	0.2
	Ru-O2	4.2	2.01	0.004		
$Re_{0.06}Ru_{0.94}O_2$ after OER[b]	Ru-O1	1.8	1.92	0.003	6.6	0.2
	Ru-O2	4.1	2.01	0.004		
$Re_{0.06}Ru_{0.94}O_2$ OCP[b]	Ru-O1	1.8	1.94	0.003	6.1	0.2
	Ru-O2	3.9	2.00	0.004		
$Re_{0.06}Ru_{0.94}O_2$ 1.2 V[b]	Ru-O1	1.8	1.93	0.003	8.5	0.1
	Ru-O2	3.8	2.00	0.004		
$Re_{0.06}Ru_{0.94}O_2$ 1.3 V[b]	Ru-O1	1.8	1.92	0.003	8.7	0.1
	Ru-O2	3.8	2.00	0.004		
$Re_{0.06}Ru_{0.94}O_2$ 1.4 V[b]	Ru-O1	1.8	1.92	0.003	8.5	0.2
	Ru-O2	3.8	2.00	0.004		
$Re_{0.06}Ru_{0.94}O_2$ 1.5 V[b]	Ru-O1	1.8	1.91	0.003	8.5	0.1
	Ru-O2	4.0	2.01	0.004		
$Re_{0.06}Ru_{0.94}O_2$ 1.6 V[b]	Ru-O1	1.8	1.91	0.003	9.0	0.1
	Ru-O2	3.9	2.01	0.004		

[a]*N*, coordination number; *R*, distance between absorber and backscatter atoms; σ^2, Debye–Waller factor to account for both thermal and structural disorders; ΔE_0, inner potential correction; R_f factor (%) indicates the goodness of the fit. Error bounds (accuracies) that characterize the structural parameters obtained by EXAFS spectroscopy were estimated as $N \pm 20\%$; $R \pm 1\%$; $\sigma^2 \pm 20\%$; $\Delta E_0 \pm 20\%$. S_0^2 was fixed to 1.0 as determined from RuO_2 reference fitting. Ru-O1 and Ru-O2 represent the first and second nearest neighbor coordination O atoms. Bold numbers indicate fixed coordination number (*N*) according to the crystal structure. [b]Fitting range: $2.5 \le k$ (/Å) ≤ 10.7 and $0.8 \le R$ (Å) ≤ 2.3.

Supplementary Table 4 | Re L_3-edge EXAFS curve fitted parameters for $Re_{0.06}Ru_{0.94}O_2$.[a]

**Sample**	**shell**	***N***	***R* (Å)**	**σ^2 (Å^2)**	**ΔE_0 (eV)**	**R_f, %**
$Re_{0.06}Ru_{0.94}O_2$ Pristine[b]	Re-O1<br>Re-O2	3.8<br>1.9	1.82<br>1.93	0.004<br>0.005	7.8	0.6
$Re_{0.06}Ru_{0.94}O_2$ after OER[b]	Re-O1<br>Re-O2	3.9<br>1.8	1.81<br>2.00	0.004<br>0.005	6.7	0.3
$Re_{0.06}Ru_{0.94}O_2$ OCP[b]	Re-O1<br>Re-O2	3.7<br>2.0	1.82<br>2.01	0.004<br>0.005	8.2	0.1
$Re_{0.06}Ru_{0.94}O_2$ 1.2 V[b]	Re-O1<br>Re-O2	4.1<br>1.9	1.82<br>2.03	0.004<br>0.005	7.9	0.7
$Re_{0.06}Ru_{0.94}O_2$ 1.3 V[b]	Re-O1<br>Re-O2	3.4<br>1.9	1.80<br>1.97	0.004<br>0.005	6.8	0.6
$Re_{0.06}Ru_{0.94}O_2$ 1.4 V[b]	Re-O1<br>Re-O2	3.9<br>1.9	1.79<br>1.98	0.004<br>0.005	6.3	0.6
$Re_{0.06}Ru_{0.94}O_2$ 1.5 V[b]	Re-O1<br>Re-O2	4.7<br>1.2	1.81<br>2.06	0.004<br>0.005	6.8	0.3
$Re_{0.06}Ru_{0.94}O_2$ 1.6 V[b]	Re-O1<br>Re-O2	3.6<br>1.9	1.81<br>1.96	0.004<br>0.005	7.5	0.4

[a]*N*, coordination number; *R*, distance between absorber and backscatter atoms; σ^2, Debye–Waller factor to account for both thermal and structural disorders; ΔE_0, inner potential correction; R_f factor (%) indicates the goodness of the fit. Error bounds (accuracies) that characterize the structural parameters obtained by EXAFS spectroscopy were estimated as $N \pm 20\%$; $R \pm 1\%$; $\sigma^2 \pm 20\%$; $\Delta E_0 \pm 20\%$. S_0^2 was fixed to 0.75 as determined from Re^{7+} aq. reference fitting. Re-O1 and Re-O2 represent the first and second nearest neighbor coordination O atoms. Bold numbers indicate fixed coordination number (*N*) according to the crystal structure. [b]Fitting range: $1.8 \le k$ (/Å) ≤ 9.0 and $0.8 \le R$ (Å) ≤ 2.3.

Supplementary Note 1

Turnover frequency (TOF)

TOF for catalysts was measured in a three-electrode system with glassy carbon as the working electrode. TOF is defined as the frequency of reaction per active site, to compare the intrinsic activity of different catalysts. TOF was computed from:

$$\mathrm{TOF}\ (\mathrm{O_2\ h^{-1}}) = 3600 \times \frac{1\ \mathrm{C\ s^{-1}}}{1\ \mathrm{A}} \times \frac{1\ \mathrm{mol}}{96{,}485\ \mathrm{C}} \times \frac{1\ mol_{\ oxygen}}{4\ mol_{\ electron}} \times \frac{6.023\times10^{23}}{1\ mol_{\ oxygen}} \times \frac{1}{\text{Number of Ru active sites}} \qquad \text{(S1)}$$

$$\text{Number of Ru active sites} = \frac{\text{Catalysts loading mass} \times \text{Ru mass percentage} \times 6.023\times10^{23}}{101\ \mathrm{g\ mol^{-1}}} \qquad \text{(S2)}$$

All Ru atoms were assumed as active sites, computed based on ICP-MS findings.

Supplementary Note 2

Faradaic efficiency (FE)

FE was computed from:

$$\mathrm{FE}\ (\%) = \frac{\text{Experimental } \mathrm{O_2}}{\text{Theoretical } \mathrm{O_2}} \times 100 \qquad \text{(S3)}$$

Theoretical O_2 was computed from:

$$\text{Theoretical } \mathrm{O_2} = \frac{\mathrm{I\times t}}{4\times96485} \qquad \text{(S4)}$$

Atmospheric O_2 was determined via fluorescence probe (Ocean Insight, FOSPOR-R, drift at 0% O_2, 0.0003% h^{-1}). Prior to testing, the probe was calibrated by a two-point calibration method. The experimental amount of O_2 gas was determined from:

$$\text{Experimental } \mathrm{O_2} = \frac{\mathrm{V\times P}}{22.4} \qquad \text{(S5)}$$

where V is the volume of the headspace of the cell and P percentage of O_2 in the headspace.

Supplementary Note 3

Mass activity computation

The Mass activity for catalysts was measured based on data obtained from the three-electrode system with glassy carbon as the working electrode. Mass activity is used to compare the intrinsic activity of different catalysts. Mass activity was computed from:

$$\text{Number of Ru active sites} = \frac{\text{Current density (A cm}^{-2}\text{)}}{\text{Ru loading mass (g cm}^{-2}\text{)}} \quad \text{(S6)}$$

All Ru atoms were assumed as active sites, which were computed based on ICP-MS findings.

Supplementary Note 4

Specific area activity

The specific area activity was determined by normalizing the ECSA for different catalysts. The specific current density per ECSA (j_a) was computed from:

$$j_a = \frac{j_{geo} \times A_{geo} \times C_s}{C_{dl}} \quad \text{(S7)}$$

where j_{geo} is the geometric area current density and A_{geo} the geometric area of the glassy carbon electrode (0.19625 cm^{-2}). C_{dl} was measured from CV in **Supplementary Fig. 11**. The C_s for 0.035 mF cm^{-2} was used to estimate ECSA.

Supplementary Note 5

Loss computation

The loss of catalysts during stability was computed from:

$$\text{Loss percentage (\%)} = \frac{\text{Dissolved metal concentration} \times \text{Electrolyte volume}}{\text{Mass of metal in catalysts}} \times 100\% \quad \text{(S8)}$$

where Dissolved metal concentration is obtained *via* ICP-MS, the electrolyte volume is 40 mL. The Mass of metal in catalysts is computed based on the ratio of metal in the catalyst. For example for RuO_2, the loading catalyst on working electrode (1 cm^{-2}) is 0.2 mg. The Ru loading mass is 0.2 ×0.76 = 0.152 mg.

Supplementary Note 6

Stability number

The stability number of catalysts (S-number) was computed from:

$$\text{Stability number} = \frac{\text{Number of } O_2}{\text{Number of dissolved Ru}} \tag{S9}$$

where N_{oxygen} is the molar number of total oxygen evolved within a period of time (computed from total charge). The total charge is obtained from t×I. Because the Faradaic efficiency is 100 %, the molar number of O_2 in 200 h is 0.0187 mol. The total dissolved noble metal number measured *via* ICP-MS.

Supplementary Note 7

Cost of catalyst

The cost of catalyst is computed based on price of noble metal only. For 2022, the cost for Ir is US \$176 g^{-1}, Ru is US \$22 g^{-1} and Re US \$1.7 g^{-1}. Therefore, the cost for $Re_{0.06}Ru_{0.94}O_2$ is ~ US\$15.3 g^{-1} (based on Ru wt% and Re wt%), which is a magnitude less than for commercial IrO_2 of ~ US\$151 g^{-1}.

Supplementary References

1 Lin, C. *et al.* In-situ reconstructed Ru atom array on α-MnO_2 with enhanced performance for acidic water oxidation. *Nat. Catal.* **4**, 1012-1023 (2021).

2 Retuerto, M. *et al.* Na-doped ruthenium perovskite electrocatalysts with improved oxygen evolution activity and durability in acidic media. *Nat. Commun.* **10**, 2041 (2019).

3 Miao, X. *et al.* Quadruple perovskite ruthenate as a highly efficient catalyst for acidic water oxidation. *Nat. Commun.* **10**, 3809 (2019).

4 Lin, Y. *et al.* Chromium-ruthenium oxide solid solution electrocatalyst for highly efficient oxygen evolution reaction in acidic media. *Nat. Commun.* **10**, 162 (2019).

5 Ge, R. *et al.* Ultrafine defective RuO_2 electrocatalyst integrated on carbon cloth for robust water oxidation in acidic media. *Adv. Energy Mater.* **9**, 1901313 (2019).

6 Su, J. *et al.* Assembling ultrasmall copper-doped ruthenium oxide nanocrystals into hollow porous polyhedra: highly robust electrocatalysts for oxygen evolution in acidic media. *Adv. Mater.* **30**, 1801351 (2018).

7 Wen, Y. *et al.* Stabilizing highly active Ru sites by suppressing lattice oxygen participation in acidic water oxidation. *J. Am. Chem. Soc.* **143**, 6482-6490 (2021).

8 Hao, S. *et al.* Torsion strained iridium oxide for efficient acidic water oxidation in proton exchange membrane electrolyzers. *Nat. Nanotech.* **16**, 1371-1377 (2021).

9 Dang, Q. *et al.* Iridium metallene oxide for acidic oxygen evolution catalysis. *Nat. Commun.* **12**, 6007 (2021).

10 Zhang, J. *et al.* Iridium nanoparticles anchored on 3D graphite foam as a bifunctional electrocatalyst for excellent overall water splitting in acidic solution. *Nano Energy* **40**, 27-33 (2017).

11 Ghadge, S. D. *et al.* Experimental and theoretical validation of high efficiency and robust electrocatalytic response of one-dimensional (1D) $(Mn,Ir)O_2$:10F nanorods for the oxygen evolution reaction in PEM-based water electrolysis. *ACS Catal.* **9**, 2134-2157 (2019).

12 Wang, J. *et al.* Single-site Pt-doped RuO_2 hollow nanospheres with interstitial C for high-performance acidic overall water splitting. *Sci. Adv.* **8**, eabl9271 (2022).

13 Liu, S. *et al.* Ultrathin perovskite derived Ir-based nanosheets for high-performance electrocatalytic water splitting. *Energy Environ. Sci.* **15**, 1672-1681 (2022).

14 Shan, J., Ling, T., Davey, K., Zheng, Y. & Qiao, S.-Z. Transition-metal-doped RuIr bifunctional nanocrystals for overall water splitting in acidic environments. *Adv. Mater.* **31**, 1900510 (2019).

15 Shan, J. *et al.* Charge-redistribution-enhanced nanocrystalline Ru@IrO_x electrocatalysts for oxygen evolution in acidic media. *Chem* **5**, 445-459 (2019).

16 Shan, J. *et al.* Short-range ordered iridium single atoms integrated into cobalt oxide spinel structure for highly efficient electrocatalytic water oxidation. *J. Am. Chem. Soc.* **143**, 5201-5211 (2021).

17 Yao, Y. *et al.* Engineering the electronic structure of single atom Ru sites via compressive strain boosts acidic water oxidation electrocatalysis. *Nat. Catal.* **2**, 304-313 (2019).

18 Cao, L. *et al.* Dynamic oxygen adsorption on single-atomic ruthenium catalyst with high performance for acidic oxygen evolution reaction. *Nat. Commun.* **10**, 4849 (2019).
